# An online parenting intervention to prevent affective disorders in high-risk adolescents: the PIPA trial protocol

**DOI:** 10.1186/s13063-022-06563-8

**Published:** 2022-08-15

**Authors:** C. Connor, Yap MBH, J. Warwick, M. Birchwood, N De Valliere, J. Madan, G. A. Melvin, E. Padfield, P. Patterson, S. Petrou, K. Raynes, S. Stewart-Brown, A. Thompson

**Affiliations:** 1 University of Warwick, Gibbet Hill Road, Coventry, CV4 7AL UK; 2 Monash University, Melbourne, Australia; 3 Birmingham Women’s & Children’s NHS Foundation Trust, Birmingham, UK

**Keywords:** Randomised controlled trial, Internet, Online parenting programme, Prevention, Depression and anxiety, Adolescence

## Abstract

**Background:**

Adolescent depression can place a young person at high risk of recurrence and a range of psychosocial and vocational impairments in adult life, highlighting the importance of early recognition and prevention. Parents/carers are well placed to notice changes in their child’s emotional wellbeing which may indicate risk, and there is increasing evidence that modifiable factors exist within the family system that may help reduce the risk of depression and anxiety in an adolescent. A randomised controlled trial (RCT) of the online personalised ‘Partners in Parenting’ programme developed in Australia, focused on improving parenting skills, knowledge and awareness, showed that it helped reduce depressive symptoms in adolescents who had elevated symptom levels at baseline. We have adapted this programme and will conduct an RCT in a UK setting.

**Methods:**

In total, 433 family dyads (parents/carers and children aged 11–15) will be recruited through schools, social media and parenting/family groups in the UK. Following completion of screening measures of their adolescent’s depressive symptoms, parents/carers of those with elevated scores will be randomised to receive either the online personalised parenting programme *or* a series of online factsheets about adolescent development and wellbeing. The primary objective will be to test whether the personalised parenting intervention reduces depressive symptoms in adolescents deemed at high risk, using the parent-reported Short Mood & Feelings Questionnaire. Follow-up assessments will be undertaken at 6 and 15 months and a process evaluation will examine context, implementation and impact of the intervention. An economic evaluation will also be incorporated with cost-effectiveness of the parenting intervention expressed in terms of incremental cost per quality-adjusted life year gained.

**Discussion:**

Half of mental health problems emerge before mid-adolescence and approximately three-quarters by mid-20s, highlighting the need for effective preventative strategies. However, few early interventions are family focused and delivered online. We aim to conduct a National Institute for Health and Care Research (NIHR) funded RCT of the online personalised ‘Partners in Parenting’ programme, proven effective in Australia, targeting adolescents at risk of depression to evaluate its effectiveness, cost-effectiveness and usability in a UK setting.

**Trial registration {2a}:**

ISRCTN63358736. Registered 18 September 2019.

**Supplementary Information:**

The online version contains supplementary material available at 10.1186/s13063-022-06563-8.

## Administrative information

Note: the numbers in curly brackets in this protocol refer to SPIRIT checklist item numbers. The order of the items has been modified to group similar items (see http://www.equator-network.org/reporting-guidelines/spirit-2013-statement-defining-standard-protocol-items-for-clinical-trials/.)

**Table:** 

Title {1}	An online Parenting Intervention to Prevent affective disorders in high-risk Adolescents: The PIPA Trial Protocol
Trial registration {2a and 2b}	ISRCTN63358736
Protocol version {3}	5^th^ January 2022 – Version 20
Funding {4}	National Institute for Health and Care Research Public Health Research NIHR (17/04/34)
Author details {5a}	Connor C*/Yap M B H**, Warwick J*, Birchwood M*, De Valliere K N*, Madan J*, Melvin GA**, Padfield E*, Patterson P, Petrou S*, Raynes K*, Stewart-Brown S* & Thompson A* *University of Warwick ** Monash University ***Birmingham Women’s & Children’s NHS Foundation Trust
Name and contact information for the trial sponsors {5b}	University of Warwick Gibbet Hill Road Coventry CV4 7AL Email: sponsors@warwick.ac.uk
Role of sponsor {5c}	The University of Warwick will act as research sponsor for the trial, oversight provided by Warwick Clinical Trials Unit who will report to the Sponsorship and Oversight Committee for oversight purposes.

### Background {6a}

Approximately half of lifetime mental disorders emerge before mid-adolescence and three-quarters by mid-20s [1]. Annual mental health treatment costs in the UK are estimated to increase to over £10 billion by 2026, with costs highest in the young [2]. Depression in adolescence is of particular concern [3]; approximately one in seven (14.4%) young people aged 11–16 have a mental health disorder, with almost one in ten experiencing an emotional disorder [4].

Adolescent depression and anxiety puts young people at risk of developing more severe affective disorders [4, 5], long-term psychosocial and vocational impairments, and increased risk of suicide [6, 7] as they mature, even when symptoms are sub-threshold [8]. Early identification and indicated interventions for those with low-level symptoms have been associated with positive outcomes; with meta-analyses showing a typical reduction in depression incidence of around 20% in the 3–24 months post-intervention [9, 10].

The development and implementation of preventative strategies for adolescents is a global priority [11]. In the UK, a government green paper and associated policy documents [12] highlight adolescence as a ‘critical period’ during which preventative strategies may be at their most effective.

Robust evidence delineating risk and protective factors associated with the development of adolescent depression and anxiety [13] provides a solid foundation for public health initiatives. Focusing on these potentially malleable psychosocial factors may improve outcomes for adolescents [14]. Strategic settings for initiatives have included schools, digital/print media and the internet but interventions are typically structured, face-to-face psychological therapies, predominantly targeted towards the adolescent themselves. Whilst such initiatives have demonstrated continued efficacy at 12 months post-intervention [15], they rarely include a family component [14, 16]. A recent meta-analysis has suggested, however, that explicitly targeting parents/carers of children aged 0–18 years can produce lasting benefits for internalising outcomes, with long-term effects on anxiety (up to 11 years post intervention) and depression (up to 5.5 years). Pooled effects suggested numbers-needed-to-treat (NNT) of 10 and 11 for preventing anxiety and depression respectively [17], figures similar to interventions directly targeting adolescents (NNT=11 [15];).

The present trial, therefore, focuses on the family setting, mindful of the need to empower parents/carers and equip them with a range of skills and knowledge with which to support their child’s emotional wellbeing. The close proximity of the family unit means that parents/carers are ideally placed to notice changes in their child’s emotional wellbeing and likely to have primary responsibility for initiating help-seeking and providing support [18]. However, a national survey of Australian parents revealed their knowledge about practical ways to help reduce their child’s risk of depression is less than optimal [19], and whilst intrinsically motivated to take action for their child’s wellbeing, this lack of awareness and skills may reduce their capacity to do so [20].

Despite global mental health policies and action plans prioritising preventative youth mental health strategies [12, 21, 22], there remains a gap in the provision of *evidence-based family resources* to upskill and improve knowledge and awareness of the risk and protective factors under parents’/carers’ direct control and influence [23]. Protective factors can be as fundamental as encouraging healthy sleep, diet and engaging in physical activity [24]. However, there is a strong evidence base for three key parental protective factors (*warmth*, *autonomy granting* and *monitoring*) and three risk factors (*inter-parental conflict*, *over-involvement* and *aversiveness* [25]). Such factors are akin to the concept of expressed emotion, wherein familial emotional climate and dialogical styles have been associated with psychosis relapse [26], poor behavioural and social outcomes for adolescents [27]. Most evidence-based targeted parenting interventions, however, are face-to-face, group programmes delivered by trained professionals. This can make them expensive and susceptible to a host of engagement barriers such as scheduling and privacy concerns, thereby limiting their public health benefit [28, 29]. Furthermore, whilst group programmes may offer social support [30], many families may prefer a private space to explore personal issues [17].

Digital technology offers such confidentiality and may be viewed as less stigmatising [28], more flexible and accessible [31]. Web-based programmes also offer greater potential for personalisation, screening individuals across a range of empirically derived risk and protective factors to target in an intervention [20], thereby ensuring thorough coverage of significant areas of importance and breadth without imposing unnecessary burden by the addition of less personally relevant topics [20], enhancing perceived relevance [32], effectiveness [33], scalability and sustainability [32]. Such tailored online programmes are viewed favourably by parents/carers [34], with positive effects observed in terms of relationships and behaviour [35].

Online personalised parenting programmes, therefore, may be a more efficacious and economically viable public health approach to prevention [36]. Yet few researchers are fully embracing digital tools for this purpose [37]. Indeed, a recent systematic review identified only one tailored web-based parenting intervention to prevent depression and anxiety disorders in adolescents—‘Partners in Parenting’ [20, 38]. The Australian programme targets up to nine modifiable parental domains, established through systematic review and meta-analysis of modifiable parenting factors associated with adolescent depression and anxiety [24] and the consensus of international experts [25]. A randomised controlled trial (RCT) of this programme, with 359 Australian parent-adolescent dyads, found greater improvement in desired parenting techniques in the personalised programme group at post-intervention, compared to an active control (Cohen’s *d*=0.51). Among adolescents with elevated depressive symptoms at baseline (*n*=105), the personalised programme group showed greater depression symptom reduction [39].

We have adapted the Australian online ‘Partners in Parenting’ programme for use in a UK setting and will conduct an RCT to test its effectiveness, cost-effectiveness and usability—‘An online *P*arenting *I*ntervention to *P*revent affective disorders in high-risk *A*dolescents: The PIPA Trial’.

### Objectives {7}

#### Primary objective

- To test the effect of an online personalised parenting programme (relative to an active control) on severity of depressive symptoms in adolescents at high risk of developing affective disorders in the UK.

#### Secondary objectives

- To test the effect of an online personalised parenting programme (relative to active control) on parenting behaviour, self-efficacy and mental wellbeing in parents/carers.
- To test the effect of an online personalised parenting programme (relative to active control) on emotional regulation, anxiety symptoms, emotional and behavioural difficulties and quality of life in adolescents.
- To evaluate the cost-effectiveness of an online personalised parenting programme.

### Trial design {8}

The PIPA trial is a two-arm, superiority, intention to treat RCT (allocation ratio 1:1). Parents/carers will be randomised to either the personalised parenting programme (intervention) or standard educational package (active control). Both will be delivered online and *not* by researchers. There will be three measurement time-points: baseline, 6 months post-randomisation and 15 months post-randomisation.

**Fig. 1:**
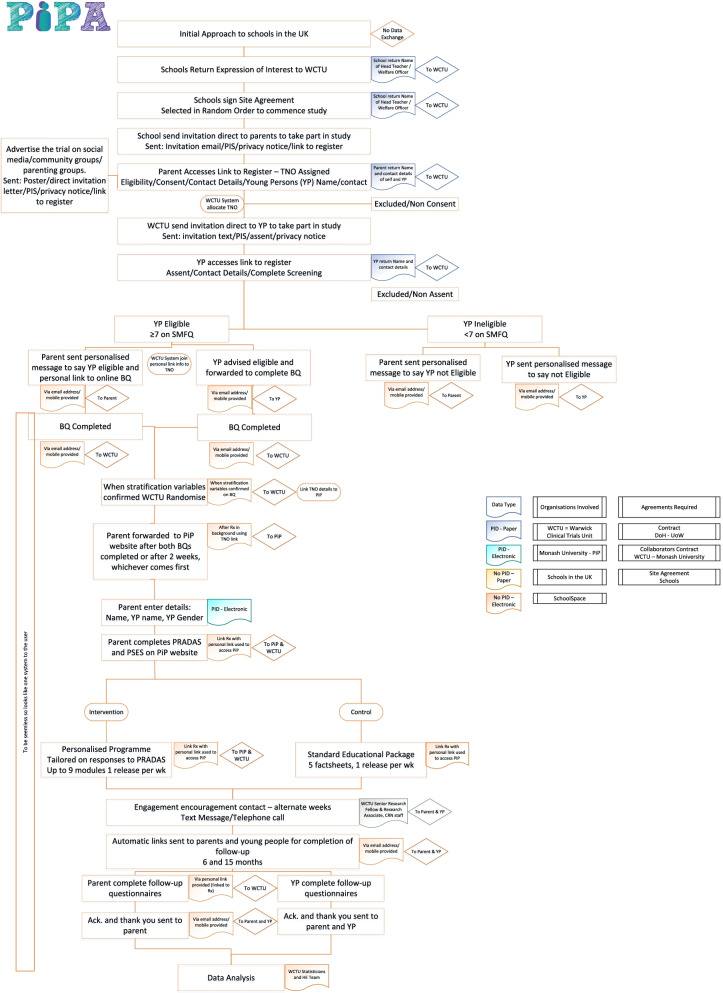
Trial flow diagram

The research design includes a health economic evaluation to establish cost-effectiveness. A process evaluation will also explore participant experience to determine acceptability and usability of the programme by conducting a series of focus groups with parents/carers and teachers and in-depth interviews with family dyads (parents/carers and adolescents). A flow diagram of the trial is shown in Fig. 1.

## Methods

### Study setting {9}

The mid-year 2020 population of the UK was estimated to be 67.1 million, with around 6% (4 million) aged 11–15years [40]. Participants will be recruited through secondary schools in the UK. There are 4190 secondary schools in the UK and 4.1 million secondary school pupils [41].

Participants will also be recruited directly via social media (e.g. Facebook and Twitter) and parenting/family and other community groups in the UK. In January 2021, there were an estimated 53 million active social media users in the UK with an average of 1 h and 49 min spent using social media daily [42].

### Eligibility criteria {10}

Participants will confirm eligibility for the trial, with some data checks conducted by the trial team. Inclusion and exclusion criteria are detailed in Table 1.

**Table 1:** Inclusion and exclusion criteria

	Parent/carer	Young person
**Inclusion criteria**	• Age ≥18 years • Biological parent/carer/non-biological parent/grandparent/legal guardian • Access to the internet and personal email account • Mobile phone number	• Aged 11–15 years • Parent/carer has given informed consent • Has a reading age of 11+ years • Access to mobile phone and internet • Living with participating parent/carer • Score 7 or above on Short Mood and Feelings Questionnaire (SMFQ)
**Exclusion criteria**	• Unable to access the PIPA database and/or Partners in Parenting website • Previous failed screening or randomisation in present trial • Participation in another parenting intervention in last 90 days	

### Who will take informed consent {26a}

Parents/carers will provide electronic consent for their own *and* their child’s participation via the PIPA trial website. Adolescents will be sent a text to confirm assent. Consent/assent forms will be held electronically at Warwick Clinical Trials Unit (WCTU), following WCTU Data Management and Security Standard Operating Procedures (SOPs).

### Additional consent provisions for collection and use of participant data and biological specimens {26b}

Consent/assent procedure includes the option for participants to permit any information collected to be used or shared anonymously with other researchers and to support future research.

## Intervention

### Explanation for the choice of comparators {6b}

The active control consists of five factsheets providing information to parents/carers about adolescent development representative of UK health promotion resources. We have chosen an active control in order to engage parents and aid retention for the duration of the trial.

### Intervention description {11a}

#### Personalised programme

The personalised programme is delivered online via the ‘Partners in Parenting’ website [20]. The programme fulfills the principles of the evidence-based Persuasive Systems Design model, which uses technology to influence behaviour change [43]. Principles include tailoring, feedback, personalisation and self-monitoring, all associated with greater programme adherence [44]. Adaptations for a UK context were made following feedback from a series of adaptation focus groups with parents/carers, teachers and adolescents.

**Fig. 2:**
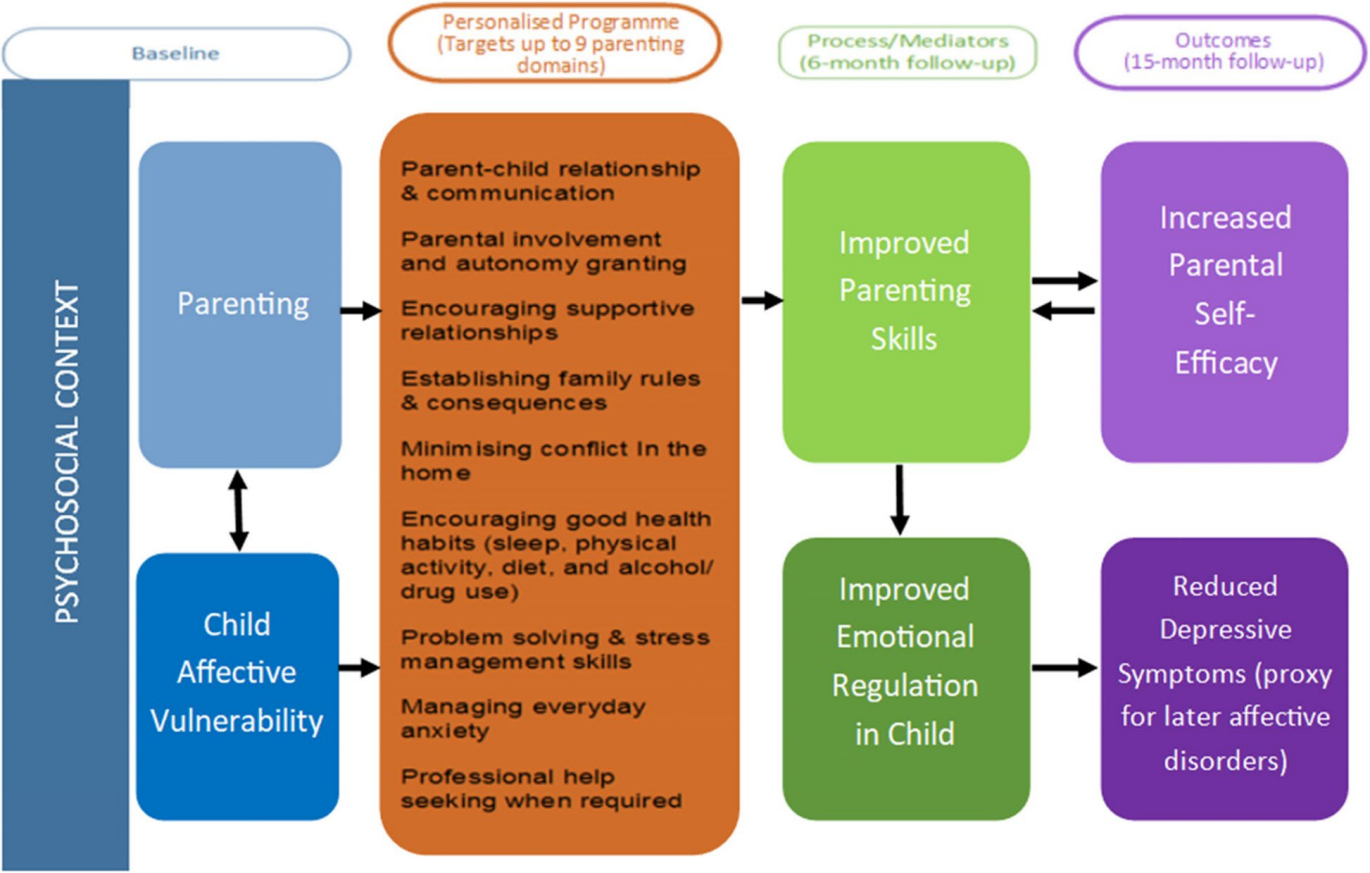
Logic model

After assessment of current parenting practices using the ‘Parenting to Reduce Adolescent Depression and Anxiety Scale’ (PRADAS [45]) and the ‘Parental Self-Efficacy Scale’ (PSES [46]) parents/carers will receive individually tailored feedback and will be recommended up to nine modules (targeting the nine modifiable parenting domains) (see Fig. 2).

Participants may accept recommended modules *or* select their own, with one module released per week until all selected have been released. Modules offer a range of parenting tips and information on adolescent mental health and wellbeing using illustrations, video and audio clips, vignettes, goal-setting exercises and ‘end-of-module’ quizzes (with immediate feedback to consolidate learning).

#### Standard educational package

The active control is also delivered via the ‘Partners in Parenting’ website and consists of five factsheets (to match expected mean number of modules received by the intervention group; *n* = 5.2 [39]) *without* tailored, actionable strategies for parents/carers and released in a set order on a weekly basis. Factsheets were adapted from highly credible resources in Australia (Raising Children [47], as used by the Australian RCT. They were modified for a UK context following feedback from adaptation focus groups with parents/carers and teachers. Factsheets cover the following topics: (1) Teen development, (2) The teenager’s developing brain, (3) The teenager’s changing body, (4) Resilience, and (5) Happy teenagers and teenage wellbeing.

### Criteria for discontinuing or modifying allocated interventions {11b}

The Participant Information Leaflet states that neither participants nor the research team will be able to choose or influence group allocation.

Parents/carers and adolescents may withdraw from the trial and/or interventions at any time without prejudice, with the option to volunteer a reason for withdrawal. Complete withdrawal will also withdraw the corresponding family member, who will be notified of this. Unless consent is *explicitly* withdrawn, participants will be followed up and data collected as per protocol until the end of the trial. Withdrawal forms will be completed by the PIPA trial team. All data up to the point of withdrawal will be retained unless requested otherwise.

### Strategies to improve adherence to interventions {11c}

Every 7 days after the intervention start date, an automated email will invite parents/carers in both groups to access their next module/factsheet, until all have been released. All participants will receive fortnightly check-in calls from research staff, following entry into the trial, alternated with text messages. These check-in calls will *not* deliver parenting advice but will be used to maintain engagement, address basic trial-related questions, encourage progression and enhance adherence.

### Relevant concomitant care permitted or prohibited during the trial {11d}

Participation in another parenting intervention will exclude individuals from participating in the current trial.

### Provisions for post-trial care {30}

A list of helpful resources will be available on the PIPA Trial website, should families require further support regarding mental health and wellbeing.

### Outcomes {12}

#### Primary outcome

- A change in parent/carer-reported adolescent depression score between entry to trial and 15 months post-randomisation (Short Mood & Feelings Questionnaire – Parent reported; SMFQ-PR [48]).

#### Secondary outcomes

Parents/carers

- Parenting behaviour (Parenting to Reduce Adolescent Depression and Anxiety Scale (PRADAS [45] and Parenting Self-Efficacy Scale (PSES) [46]) between entry to trial and 15 months post-randomisation.
- Parental wellbeing (Warwick-Edinburgh Mental Wellbeing Scale short form; SWEMWBS [49] between entry to trial and 15 months post-randomisation.
- Attachment (adapted version of the Inventory of Parent & Peer Attachment; IPPA ( [50]; JP Allen, pers. comm., 2013) between entry to trial and 15 months post-randomisation.
- Parent-reported emotional and behavioural difficulties of child (Strengths & Difficulties Questionnaire; SDQ [51]) between entry to trial and 15 months post-randomisation.
- Service Use (adapted version of the Client service receipt inventory; CSRI).
- Health-related quality of life (EuroQol 5 Dimensions 5 Level; EQ-5D-5L [52]).
- Intervention satisfaction (Intervention Satisfaction Survey).

Adolescents

- Emotional regulation (Difficulties in Emotional Regulation Scale Short Form; DERS-SF [53]) between entry to trial and 15 months post-randomisation.
- Depression (Development & Wellbeing Assessment depression component; DAWBA [54]) between entry to trial and 15 months post-randomisation.
- Anxiety (Children’s Anxiety Scale – 8 items; CAS-8 [55]) between entry to trial and 15 months post-randomisation.
- Attachment (Inventory of Parent & Peer attachment—Revised for Children; IPPA-R [56]) between entry to trial and 15 months post-randomisation.
- Emotional and behavioural difficulties (SDQ [51, 57]) between entry to trial and 15 months post-randomisation.
- Health-related quality of life (Childhood Health Utility Index 9D (CHU-9D) [58]; EuroQol 5 Dimensions 5 Level Youth, EQ-5D-5L-Y and EQ-5D-5L-Y proxy [52, 59].

### Participant timeline {13}

A timeline of assessments for participants is shown in Table 2. All assessments will be completed via the PIPA database.

**Table 2:** Assessments and timeline for participants

Time-point	Baseline		6 months (post-randomisation)		15 months (approx. 12 months post-randomisation)	
	Parent/Carer	Young Person	Parent/Carer	Young Person	Parent/Carer	Young Person
**Pre-entry into trial**						
Participant details	**✓**					
Inclusion/exclusion criteria	**✓**					
Informed consent	**✓**					
Assent		**✓**				
Screening^a^		**✓**^a^				
**Trial**						
Medical history	**✓**					
SMFQ (primary outcome)^a^	**✓**	**✓**^a^	**✓**	**✓**	**✓**	**✓**
PRADAS	**✓**		**✓**		**✓**	
IPPA	**✓**	**✓**	**✓**	**✓**	**✓**	**✓**
PSES	**✓**		**✓**		**✓**	
DERS-SF		**✓**		**✓**		**✓**
Short WEMWBS	**✓**		**✓**		**✓**	
CAS-8		**✓**		**✓**		**✓**
SDQ	**✓**	**✓**	**✓**	**✓**	**✓**	**✓**
DAWBA		**✓**				**✓**
CHU-9D		**✓**		**✓**		**✓**
EQ-5D-5L-Y		**✓**		**✓**		**✓**
EQ-5D-5L	**✓**		**✓**		**✓**	
EQ5D-5L-Y proxy	**✓**		**✓**		**✓**	
Client service receipt inventory	**✓**		**✓**		**✓**	
Intervention satisfaction survey			**✓**		**✓**	

^a^SMFQ at baseline will be collected during screening for young person

#### Adolescents

Following assent, adolescents will complete the SMFQ [48].

#### Parents/carers

SMFQ data from a large population samples suggest that more than 20% of adolescents will report depressive symptoms [60]. Parents/carers of adolescents scoring ≥7 (indicative of elevated risk of depression and/or depressive disorder [61, 62]) will receive an automated message confirming eligibility. Families of those scoring <7 will be deemed ineligible and receive an automatically generated email or text informing them of this. Should they require further information or guidance, they will be directed to the list of helpful resources available on the PIPA trial website.

Whilst multiple parents/carers from the same family may participate in the programme, only one will be asked to complete baseline and follow-up measures. Baseline assessment of parenting practices (using PRADAS and PSES) will assess the nine modifiable parenting domains (see Fig. 2). Participants will then be randomly allocated to receive either the personalised programme or standard educational package.

### Sample size {14}

In the Australian RCT [39], a reduction in depressive symptoms (from pre- to post-intervention) in the subsample of adolescents (*n*=105) with elevated baseline depressive symptoms was greater in the intervention group compared to the active control (effect size 0.35; Cohen’s *d*, *n*=105). Assuming the correlation between pre- and post-intervention SMFQ scores is 0.5, 346 families would provide 90% power to detect a difference of 0.35 in the primary outcome between trial arms at the 5% level. To allow 20% loss to follow-up, the total family recruitment target is 433, enabling sufficient numbers for sub-group analyses (for example, young people from low socioeconomic status and/or BME backgrounds).

### Recruitment {15}

Schools will be enlisted through existing contacts (see Acknowledgements), school consortiums, mental health and wellbeing leads and senior management teams and Local Clinical Research Networks (LCRN) across the UK. Due to the ongoing COVID-19 pandemic restrictions, all meetings with contacts will occur online via Microsoft ® Teams.

Interested schools will sign a site agreement and nominate a key member of staff to work with the research team to promote and engage families. The research team will be guided by individual schools as to the most appropriate way of engaging families. These will include distribution of promotional materials (posters/information leaflets, see Additional file 1) via school intranet, communication app and/or email. Parents/carers and young people will also be able to register directly for the trial through the wider community. Trial information and a link to the trial will be distributed via University of Warwick social media (Twitter and Facebook) and family/parenting groups and charities in the UK to allow for this. Those interested in participating will be asked to register for the trial and provide consent for their and their child’s participation on the dedicated trial database. They will also provide contact details for their child.

## Assignment of interventions

### Sequence generation {16a}

Allocation to trial arms will be implemented using WCTU’s automated minimisation procedure within the PIPA trial database. This adaptive stratified sampling method will minimise imbalance between number of participants in each intervention group [63].

### Concealment mechanism {16b}

Allocation will be concealed until selection criteria for entry into the trial have been met. This will be implemented, maintained and electronically revealed by WCTU.

### Implementation {16c}

Allocation will be 1:1 ratio between intervention and active control.

## Assignment of interventions: blinding

### Blinding {17a}

The Chief Investigator (CI) and Health Economist will be blinded after assignment to intervention. The Statistician, Assistant Professor, Research Associate, Senior Project Manager and Trial Manager (TM) *will not* be blinded to allocations.

### Procedure for un-blinding if needed {17b}

The Data Monitoring Committee (DMC) will have access to un-blinded, aggregate, comparative data. Un-blinding will not occur until all decisions on data evaluability have been documented.

## Data collection and management

### Plans for assessment and collection of outcomes {18a}

#### Measures (see Table 2)

##### Primary outcome measure

Adolescent depression

- Short Mood & Feelings Questionnaire (SMFQ [48])

The parent-report version of the SMFQ will be used as the primary outcome measure. This is a brief 13-item instrument to measure depressive symptomatology in 8–18-year-olds, demonstrating good to excellent internal consistency (Cronbach’s alpha), good reliability and validity with the clinician-rated Children’s Depression Rating Scale-Revised [64] and Reynolds Adolescent Depression Scale as well as satisfactory diagnostic accuracy and sensitivity to change. The items are rated using a 3-point Likert scale (not true = 0; sometimes true = 1; not true = 2). Example items include ‘I feel miserable or unhappy’ and ‘I cried a lot’. Scores are calculated by summing point values from each item response, with total SMFQ scores ranging from 0 to 26. The optimal cut-off value for differentiating *clinical cases* of depression is ≥12 [65]. A cut-off of ≥7 is taken to be indicative of *low-level* symptoms and elevated risk for depressive disorder [61, 62] and will be used in the trial.

Both the parent-report and young person-report versions of the SMFQ will be utilised and will be completed at all time-points. The primary outcome will be change in *parent-reported* depressive symptoms between baseline and 15 months post-randomisation. The *young person-report* version will be used to determine eligibility for the trial with a cut-off score of ≥7 and will also be used as a secondary outcome measure examining change in self-reported depressive symptoms between baseline and 15 months post-randomisation.

##### Secondary outcome measures

Parenting

- Parenting to Reduce Adolescent Depression & Anxiety Scale (PRADAS [45]) and The Parental Self-Efficacy Scale (PSES [46])

The PRADAS assesses parental concordance with a range of evidence-based parenting guidelines for the prevention of depression and anxiety across nine domains: *parent-child relationship*, *involvement*, *relationships with others*, *family rules*, *home environment*, *health habits*, *dealing with problems*, *coping with anxiety* and *professional help-seeking*. Reliability, test-retest reliability and convergent validity of the PRADAS have been supported by moderate to high correlations with established parenting measures [45].

The PSES was developed to measure parenting self-efficacy in behaviours that may reduce adolescent risk for depression and anxiety. It utilises a Likert-type scale to assess parental confidence in carrying out behaviours related to the nine domains assessed by the PRADAS and is administered in conjunction with the PRADAS. The PSES has been found to have high reliability and construct and convergent validity [46]. The PRADAS and PSES will be completed by parents/carers at baseline, 6 months and 15 months.

Parental mental wellbeing

- The Short Warwick-Edinburgh Mental Wellbeing Scale (SWEMWBS [49])

The SWEMWBS is a mental wellbeing measure for use with a wide range of populations. It consists of seven positively worded items rated on a Likert-type scale. It has shown good content validity with high test-re-test reliability (0.83 [66]). It will be completed at baseline, 6 months and 15 months.

Adolescent resilience

- The Difficulties in Emotional Regulation Scale - Short Form (DERS-SF [53])

Developed from the original DERS [67], this scale assesses emotional regulation problems in adults and adolescents. It consists of six sub-scales, each with three items: *limited access to emotion regulation strategies*, *non-acceptance of emotional responses*, *difficulty with impulse control*, *difficulty engaging in goal-directed behaviour*, *lack of emotional awareness* and *clarity*. Confirmatory analysis found similar correlation patterns to full DERS measure, ranging from 0.90 to 0.98, sharing 81–96% variance [53]. The DERS-SF will be used to assess resilience of adolescents and will be completed at baseline, 6 months and 15 months.

Adolescent anxiety

- The Children’s Anxiety Scale-8 (CAS-8 [55])

The CAS-8 is a brief screening instrument for anxiety symptoms in children. Respondents are asked to indicate how often they experience each symptom (e.g. *I worry about things*, *I feel nervous*) on a 4-point scale. It has demonstrated high internal consistency (*α*=0.92 [68]) and will be completed by adolescents at baseline, 6 months and 15 months.

Cases of adolescent depression

- The Development and Wellbeing Assessment (DAWBA [54])

The DAWBA will be used to identify likely cases of adolescent depression. It was designed to provide ICD-10 and DSM-V diagnoses for a range of mental health disorders in adolescence and is a well-used epidemiological measure [69]. It will be completed at baseline and 15 months.

Emotional and behavioural difficulties in adolescents

- The Strengths and Difficulties Questionnaire (SDQ [51, 57])

The SDQ is utilised to assess the emotional wellbeing of children and adolescents. It consists of 25 items rated on five scales (*emotional*, *conduct problems*, *hyperactivity/inattention*, *peer relationships* and *pro-social behaviour*). A 3-point Likert scale is used it indicate how much each attribute applies to the adolescent in question. The SDQ has good internal reliability. Both the parent/carer-report and adolescent-report versions will be completed at baseline, 6 months and 15 months.

Parental attachment

- The Inventory of Parent and Peer Attachment (IPPA ( [50]; JP Allen, pers. comm., 2013)) and The Inventory of Parent and Peer Attachment—Revised for Children (IPPA-R [56])

The IPPA will be utilised to measure parental attachment with adolescent. The measure consists of 25 items scored on a 5-point Likert scale and has acceptable to high Cronbach’s alphas (*α*=0.70–0.89). The IPPA-R will be completed by adolescents to measure attachment with parent/carer. The IPPA-R consists of 28 items using a 5-point Likert scale, divided into three sub-scales (*trust*, *communication*, *alienation*). It demonstrates good internal consistency across the three sub-scales (*α*=0.78–0.82). Both parent-report and young person-reported measures will be completed at baseline, 6 months and 15 months.

##### Health-related quality of life

Adolescent

- The Child Health Utility Index 9D (CHU-9D [58]) and EuroQol 5 Dimensions Youth 5 (EQ-5D-5L-Y [59])

These are generic measures of health-related quality of life in children and will be completed by adolescents at baseline, 6 months and 15 months. The CHU-9D assesses children’s functioning on the day across nine domains: *worry*, *sadness*, *pain*, *tiredness*, *annoyance*, *school*, *sleep*, *daily routine* and *activities*. The EQ-5D-5L-Y requires respondents to report their health on five different dimensions (*mobility*, *self-care*, *usual activities*, *pain/discomfort* and *anxiety/depression*) with five severity levels and rate their overall health using a visual analogue scale.

Parent/carer

- EuroQol 5 Dimensions 5 Level (EQ-5D-5L) and EQ-5D-5L-Y proxy [52]

Completed by parents/carers at baseline, 6 months and 15 months, the EQ-5D-5L is a self-report of their own health whilst the proxy measure is regarding their child’s health.

Data will be converted into health utilities using established utility algorithms to estimate family dyad quality-adjusted life years (QALYs; see Health Economic Analysis). The QALY is a measure of health outcome incorporated into cost-effectiveness analysis intended to aid decision-makers charged with allocating scarce resources across competing healthcare programmes and is required by the National Institute for Health and Care Excellence in England and Wales for health technology assessment.

Parent/carer estimates of resource utilisation

- The Client Service Receipt Inventory (CSRI)

This is a bespoke measure of health, social care and education-related resource use over the trial follow-up period. It covers *hospital care*, *community-based health and social services use*, *medication use*, *private health care*, *school absence*, *special educational needs and other support*, *time off work by parents* and *other expenses* and will be completed by parents/carers at baseline, 6 months and 15 months.

### Plans to promote participant retention and complete follow-up {18b}

Based on evidence that incentives may increase rate of assessment completion [70], each participating family will be offered a £25 voucher following completion of all baseline and follow-up assessments.

Automated weekly emails and fortnightly check-in calls, alternated with a text message, will be made by the research team to help maintain engagement. Calls will *not* offer parenting advice.

Links to the online PIPA database will be sent by email and/or text to parents/carers and adolescents for completion of follow-up assessments.

### Data management {19}

The University of Warwick will act as data controller for the trial. Informed consent and assent forms will be held electronically at WCTU following Data Management and Security SOPs.

Access to personal/confidential data will be monitored throughout the trial and restricted to those delegated roles by the CI.

Monash University will be the data processor for the trial and will maintain the ‘Partners in Parenting’ website which houses the interventions, baseline PRADAS and PSES questionnaires. All data will be stored after generation on a Google Cloud Platform, via the European Data Centre. Pseudonymised data will be transferred to the University of Warwick and stored in the same manner as other trial data.

The text and email messaging company Twilio© will be utilised to communicate with participants with access to participant names, email addresses and mobile phone numbers. Selected staff from Clinical Research Networks providing phone-call support will have limited access to these and limited intervention data in order to complete fortnightly check-in calls. Some anonymized questionnaire data will be transferred for scoring purposes. Appropriate contracts are in place for these purposes.

Storage and transfer of data throughout the trial will be done in accordance with University of Warwick data policies and standard operational procedures. WCTU will archive trial documentation and data for at least 10 years after completion of the trial.

### Confidentiality {27}

Any personal data will be handled and stored in accordance with the 2018 Data Protection Act held securely at WCTU until the end of the trial and disposed of in accordance with WCTU procedures. Participants will be given unique trial identification numbers to maintain anonymity. The need for personal details, necessary for phone calls and texts is clearly documented in participant information sheets (see Additional file 1). Personal identifying information for ineligible families and those wishing to withdraw will be deleted at the end of the trial as per WCTU SOPs.

Data will only be accessible by the PIPA research team using assigned logins and passwords. All data will be treated in confidence and not disclosed or used for any unrelated purposes (except by prior agreement with the participant or to address specified risks to the participant, researcher or others throughout the trial).

## Statistical methods

### Statistical methods for primary and secondary outcomes {20a}

All analyses will be performed on an intention-to-treat basis (ITT). The ITT population will comprise all randomised participants. Baseline characteristics will be presented using descriptive statistical methods; continuous variables will be summarised using means and standard deviations and skewed continuous variables using median and inter-quartile range. Categorical data will be summarised using frequencies and percentages. Outliers will be identified using graphical methods. Participant flow throughout the trial including numbers screened, recruited, randomised, and withdrawn will be documented using a CONSORT diagram. Means and 95% confidence intervals for mental health and wellbeing, as assessed by primary and secondary outcomes, will be summarised at baseline, 6 months and 15 months post-randomisation.

The primary outcome is change in parent-reported SMFQ score between entry to the trial and 15 months post-randomisation. This will be analysed using a linear mixed model, with school as a random effect and age group and number of participating parents as fixed effects. Cases of depression between trial entry and 15 months post-randomisation will be reported and appropriate models used to assess any difference between intervention arms. Linear mixed models will also assess impact of intervention on secondary outcomes.

### Interim analyses {21b}

There are no pre-planned interim analyses or formal rules for the full PIPA trial. The DMC will review emerging trial data and external evidence on an ongoing basis and may recommend early stopping, if appropriate, following stop/go criteria:

The study will have a pilot phase to establish the feasibility of the full PIPA trial. The stop-go decision will be made by the TMG, following consultation with the TSC and DMC 9 months from trial commencement using the following recruitment stop-go criteria as a guide:

1. Go: More than 80% of the 128 dyads within 9 months since the first randomisation (February 2021)
2. Discuss: Between 50 and 80% randomised. The trial team, the NIHR and the TSC should discuss and consider additional remedial strategies
3. Stop: Less than 50% randomised. The trial could stop for lack of interest after discussions with the TSC and the NIHR

The stop/go criteria will be subject to review by the TSC/DMC in light of Covid-19 disruptions and any amendment will be notified.

### Methods for additional analyses {20b}

#### Sub-group and adjusted analyses

Separate analysis of primary outcome and effect estimates will be conducted for school location (proxy for social deprivation), parents’/carers’ highest educational level (surrogate for socioeconomic status) and ethnicity. All analyses will be adjusted by design variables (school, age group, gender of young person, gender of parent/carer and total number of parents accessing modules/factsheets).

#### Health economic evaluation

To provide best available evidence regarding future health, education and social care commissioning, cost-effectiveness will be assessed using metrics amenable to cost-effectiveness based decision-making. Primary research methods will be followed to estimate the costs of delivering the personalised programme, including programme development, web maintenance, participant monitoring activities and follow-up/management. Broader resource utilisation will be captured through bespoke online questionnaires administered at baseline, 6 months and 15 months post-randomisation. Unit costs for health, social care and education-related resources will be derived from local and national sources and estimated, in line with best practice. Young person health-related quality of life will be measured at baseline and at each follow-up point using the CHU-9D [58], the EQ-5D-5L-Y [59] and the proxy (parent assessed) EQ-5D-5L-Y [52]. Parental health-related quality of life will be measured at baseline and at each follow-up point using the EQ-5D-5L. Responses will be converted into health utilities using established utility algorithms for the purposes of parent/carer-child dyad QALY estimation. The results will be expressed in terms of incremental cost per QALY gained. We shall use non-parametric bootstrap estimation to derive 95% confidence intervals for mean cost differences between the trial groups and to calculate 95% confidence intervals for incremental cost-effectiveness ratios [71]. A series of sensitivity analyses will be undertaken to explore the implications of uncertainty surrounding the incremental cost-effectiveness ratios. In the baseline analysis, and for each sensitivity analysis, cost-effectiveness acceptability curves will be constructed using the net benefits approach [72]. More extensive economic modelling using decision-analytic methods will extend the time horizon of the economic evaluation, drawing on best available information from the literature together with stakeholder consultations to supplement trial data. Parameter uncertainty in the decision-analytic model will be explored using probabilistic sensitivity analysis. Longer-term costs and consequences will be discounted to present values using discount rates recommended for technology appraisal in the UK [73].

#### Process evaluation

A process evaluation (PE) will be conducted to test the logic model (see Fig. 2) and obtain a clear understanding of trial functioning and engagement. A PE prior to full trial was deemed unnecessary as changes to the trial/interventions at this stage would not have been possible. We plan to conduct a series of focus groups (FGs) with parents/carers and school staff, and interviews with parent/carer and adolescent dyads. Participants will have been informed of these via the Participant Information Leaflet. All participants will also complete a satisfaction and acceptability question at 6 and 15 months to further evaluate programme experience.

#### Focus groups

We will recruit up to 20 parents/carers who participated in the PIPA trial (online personalised programme and standard educational package) and up to 20 teachers who helped recruit families.

FGs will explore context, implementation and impact of the PIPA trial using questions developed by the research team following our Logic Model (see Fig. 2 and Additional file 2). Due to COVID restrictions, all FGs will be conducted online (using Microsoft® Teams) by qualitatively trained members of the research team and recorded using an external encrypted digital recorder. FG data will be transcribed and transcriptions will be analysed using Framework methodology which is ideally suited for use with research focused on specific questions, with a limited time frame, a pre-designed sample and a priori issues [74]. Findings from this analysis will further inform the topic guide for the interview schedule.

#### Interviews

We will recruit up to 30 family dyads (parent/carer and young person) who received either the personalised programme or the standard educational package. If a young person refuses to participate, or a parent/carer does not wish for their child to take part, parents/carers may be interviewed alone, providing their child has assented to this. This will still be counted as a family dyad.

A topic guide has been designed and developed by the research team (see Additional file 3) and will be further informed by results from focus groups which may produce additional areas of exploration to be included in the interviews. Interviews will be conducted online by trained researchers via Microsoft Teams and audio recorded using an external encrypted digital recorder. Interviews will be transcribed and will be analysed using Interpretive Phenomenological Analysis (IPA), a well-respected and recognised qualitative methodology with an emphasis on convergence and divergence of individual experience [75].

### Methods in analysis to handle protocol non-adherence and any statistical methods to handle missing data {20c}

Imputation of missing elements of individual scales using multiple imputation will be considered if appropriate (>10% of overall scores are missing, ≤ 80% of individual elements are not missing, missing at random assumption holds).

### Plans to give access to the full protocol, participant-level data and statistical code {31c}

All fully anonymised individual participant data collected will be available 12 months after the main trial paper has been published or 10 years after trial closure. The trial protocol will also be provided. Requests for access should be for research purposes only and supported by a methodologically sound proposal. Data will only be shared under a formal Data Sharing Agreement and transferred in accordance with University of Warwick SOP 15 and relevant legislation. Applications should be made to the PIPA trial team (pipa@warwick.ac.uk).

## Oversight and monitoring

### Composition of the coordinating centre, Trial Steering Committee (TSC) {5d}

#### Coordinating centre

WCTU will have responsibility for overall conduct of the trial, provision of adequate indemnity and oversight of participant safety.

##### TSC

In accordance with the Trial Terms of Reference, the TSC will periodically review safety data and liaise with DMC regarding safety issues.

##### TSC Independent chair

Dr. Barbara Barrett

##### TSC Members

Ms. Anna Robinson

Dr. Mona Kanaan

Professor Mick Cooper

Mr. Layne Boyden

Ms. Farzana Kausir

### Composition of the Data Monitoring Committee, its role and reporting structure {21a}

The independent DMC will periodically review trial and safety data, determine patterns and trends and identify issues which may not be apparent on an individual case basis. They may recommend termination at any point, if appropriate, following a priori stop/go criteria (see ‘Interim Analyses {21b}’).

#### Independent Chair

Professor Cathy Creswell

#### DMC Members

Dr. Adam Brentnall

Ms. Sajda Butt

### Adverse event reporting and harms {22}

#### Duty of care procedure

The parents/carers of adolescents who score ≥20 (SMFQ - child self-report OR parent-report) at baseline, 6 months or 15 months) will be sent an email within two working days of the PIPA team receiving notification of symptom elevation and include a link to help-seeking sources and the ‘Useful Resources’ page on the PIPA website.

The parent/carer of adolescents who answer Yes to any of the following items on the DAWBA:

- *Did you talk about harming yourself or killing yourself?*
- *Did you try to harm yourself or kill yourself?*
- *Over the last 4 weeks*, *have you thought about deliberately harming or hurting yourself?*
- *Over the last 4 weeks*, *have you tried to harm or hurt yourself?*

will be contacted by phone within two working days of the PIPA team receiving notification of this information. Three phone calls will be attempted over the 2-day period. If a parent/carer is not able to be contacted or does not acknowledge receipt of the email, the research team will contact the appropriate school contact for the adolescent.

Concerns about safety and wellbeing of participants will be reported to the appropriate team member who will inform the CI/delegate who will determine the escalation process. If urgent safety measures are required, the CI/delegate shall act immediately, and in any event, no later than three calendar days from the date measures are taken, give written notice to the Biomedical and Scientific Research Ethics Committee (BSREC) and Sponsor of the measures taken and the circumstances giving rise to those measures.

Confidential medical advice will be sought from a qualified clinician within WCTU who will signpost to appropriate mental health support or services, if necessary.

Only deaths that are assessed to be caused by the trial intervention will be reported to the sponsor. Notification of death forms will be completed by the trial team upon receipt of this knowledge. The CI, or nominated WCTU clinician, will make this assessment within seven calendar days of the notification. This report will be sent to the sponsor, if deemed necessary, within one working day.

Following notification of death, the other half of the family dyad will be withdrawn from the trial.

### Frequency and plans for auditing trial conduct {23}

Trial-related documents will be made available for internal monitoring and audit activities. A Trial Risk Assessment will be conducted by the TM, Senior Project Manager, Trial Statistician, CI (or delegated representative) and WCTU Quality Assurance Team.

A Trial Monitoring Plan has been developed and agreed by the TM and TSC based on the trial risk assessment.

A data management plan has been developed and agreed by the Trial Management Group.

The trial will be audited by WCTU’s Quality Assurance team as per WCTU SOPs.

### Plans for communicating important protocol amendments to relevant parties (e.g. trial participants, ethical committees) {25}

Any protocol amendments with regard to study design, recruitment, study procedures, intervention, data collection and analysis will be communicated to the Ethical Committees and funders. Protocol amendments will be dealt with in accordance with WCTU SOPs and only be implemented after approval.

### Dissemination plans {31a}

The trial will be reported in accordance with CONSORT guidelines and reported to trial collaborators. All publications will be made available to the NIHR Journal library. The main report will be drafted by the trial coordinating team, and the final version will be agreed by the TSC before submission for publication.

The success of the trial depends heavily on collaboration with schools, school networks and education authorities. Equal credit will be given to those who have wholeheartedly cooperated and facilitated trial implementation. A results summary of the trial will be made available and shared with all collaborators via presentation and/or report. Results will also be presented at national and international conferences.

## Discussion

To the best of our knowledge, the PIPA trial is the first UK evaluation of the effectiveness and cost-effectiveness of an online personalised parenting programme for families with adolescents at risk of depression and will be the largest RCT of its kind worldwide. As the original programme was developed with Australian families in mind, the PIPA Trial has adapted some of its content and appearance to make it more suitable for a UK setting. All modifications were informed and guided by focus groups held with parents/carers and teaching staff who enabled us to adjust visual aspects of the programme and ensure that all terminology used was appropriate for a UK audience.

The SMFQ has several strengths which influenced our decision to utilise it as our primary outcome measure. It has demonstrated good discrimination of depression, particularly with adolescents [76] and is especially suited for use in community samples [77]. A significant advantage of the SMFQ lies in its brevity, comprised of only 13 items, making it especially suitable for use with adolescents and for online implementation.

Digital resources have become an increasingly popular source of mental health information for families and adolescents alike [6, 78]. Their anonymity, flexibility and accessibility make them an ideal choice for help-seeking for families who may be struggling to help and support an adolescent with depression [31], allowing for privacy and confidentiality alongside timely access to practical tools and resources for use in the ‘real world’. This may be of particular relevance during the COVID-19 pandemic during which usual social support mechanisms and networks will have been disrupted.

However, despite evidence that tailored online parenting programmes are popular and viewed favourably by families [34], they remain scarce [37]. To date, ‘Partners in Parenting’ is the only tailored web-based parenting intervention to prevent depression in adolescence that has undergone rigorous evaluation in an RCT [38]. However, the flexibility and reach that digital technology can offer may prove to be more economically viable and efficacious compared with other public health prevention strategies [36].

In conjunction with the Australian trials [38, 79, 80], the results of the PIPA trial will provide a robust evidence base with regard to the efficacy of this online personalised parenting programme, its potential for reducing depressive symptoms in adolescents in the UK, its acceptability and usability. It will also enable a cost/benefit comparison with typical face-to-face group based parenting programmes, informing policy and thus enhancing the design, and future delivery of more pragmatic resources for supporting families with adolescents at risk of depression and anxiety.

## Trial status

Protocol version 4 (05 January 2022)

Start of recruitment: 09 February 2021

Approx. date of recruitment completion: April 2023

## Supplementary Information

**Additional file 1.** Trial information leaflets (for schools, parents/carers and young people) and trial poster.

**Additional file 2.** Process evaluation focus group question frameworks.

**Additional file 3.** Process evaluation family interview question framework.

**Additional file 4.** Parent consent form and young person assent form.

## Data Availability

Fully anonymised individual participant data will be available 12 months after the main trial paper has been published and ending 10 years after trial closure. Data will only be shared under a formal Data Sharing Agreement and transferred in accordance with University of Warwick SOP 15 and relevant legislation. Applications should be made to the PIPA trial team (pipa@warwick.ac.uk).

## Abbreviations

BSREC: Biomedical and Scientific Research Ethics Committee
CAS-8: Children’s Anxiety Scale – 8 items
CHU-9D: Childhood Health Utility 9D
CI: Chief Investigator
CSRI: Client Service Receipt Inventory
DAWBA: Development & Wellbeing Assessment
DERS-SF: Difficulties in Emotional Regulation Scale - short form
DMC: Data Management Committee
EQ-5D-5L: EuroQol 5 Dimensions 5 Level
EQ-5D-5L-Y: EuroQol 5 Dimensions 5 Level Youth
FG: Focus Group
IPPA: Inventory of Parent & Peer Attachment
IPPA-R: Inventory of Parent & Peer Attachment—Revised for Children
ITT: Intention to Treat
NIHR: National Institute for Health and Care Research
NNT: Number need to treat
PIPA: An Online Parenting Intervention to prevent Affective Disorders in High-Risk Adolescents
PRADAS: Parenting to Reduce Adolescent Depression & Anxiety Scale
PSES: Parenting Self-Efficacy Scale
QALY: Quality Adjuster Life Year
RCT: Randomised control trial
SDQ: Strengths & Difficulties Questionnaire
SMFQ: Short Mood & Feelings Questionnaire
SOPs: Standard Operating Procedures
SWEMWBS: Warwick-Edinburgh Mental Wellbeing Scale - short form
TM: Trial Manager
TSC: Trial Steering Committee
WCTU: Warwick Clinical Trials Unit
