# Correction: An online parenting intervention to prevent affective disorders in high-risk adolescents: the PIPA trial protocol

**DOI:** 10.1186/s13063-022-06870-0

**Published:** 2022-10-31

**Authors:** C. Connor, M. B. H. Yap, J. Warwick, M. Birchwood, N. De Valliere, J. Madan, G. A. Melvin, E. Padfeld, P. Patterson, S. Petrou, K. Raynes, S. Stewart-Brown, A. Thompson

**Affiliations:** 1 University of Warwick, Gibbet Hill Road, Coventry, CV4 7AL UK; 2 Monash University, Melbourne, Australia; 3 Birmingham Women’s & Children’s NHS Foundation Trust, Birmingham, UK

**Correction: Trials 23, 655 (2022)**

**https://doi.org/10.1186/s13063-022-06563-8**

Following publication of the original article [1], it was reported that the family name and initials of M. B. H. Yap were erroneously transposed. The correct styling is presented in this correction article.

The original article [1] has been corrected.
