# Supplementary material for: An online parenting intervention to prevent affective disorders in high-risk adolescents: the PIPA trial protocol
Source: Trials. 2022 Aug 15;23:655. doi: 10.1186/s13063-022-06563-8 (PMC9376903; doi:10.1186/s13063-022-06563-8)
Supplement: Supplementary file 1 — Additional file 1. Trial information leaflets (for schools, parents/carers and young people) and trial poster. [file 13063_2022_6563_MOESM1_ESM.pdf]

An online Parenting Intervention to Prevent affective disorders in high-risk Adolescents: The PIPA Trial

School Information Sheet

An online parenting intervention to prevent affective disorders in high-risk adolescents: The PIPA Trial

Investigators: Professor Andrew Thompson, Professor Jason Madan & The PIPA Trial team.

Introduction

Approximately 10–25% of all school-age children have a recognisable mental health condition with 50% of such problems emerging before the age of 14. Untreated, these can lead to further problems in young adulthood, exposing young people to a wide range of risk behaviours and outcomes, however, the right support, before crises occur, may prevent ongoing problems.

Schools play an important role in educating and supporting children and young people who may be at risk, helping them develop coping skills and raising their knowledge and awareness of mental health issues. The development of networks such as The International Union of Health Promotion and Education and Schools for Health in Europe emphasises the unique position of schools and school personnel in this context.

Studies have shown the wide range of benefits that school-based programmes have for children and young people, especially when delivered by schools that operate a ‘whole school’ approach, integrating health and social and emotional well-being into the overall ethos of the school and engaging parents/carers in a ‘home-school’ partnership.

Working with, and supporting, families of children and young people has been emphasised by the UK Government in their document ‘No Health Without Mental Health’ (2011) but families often struggle to support their children with their mental and emotional wellbeing, despite being at the frontline in terms of recognising difficulties, responding, supporting and help-seeking for their child in times of need, with little, or no, help or guidance.

Who is organising and funding the trial?

The trial is being coordinated by the University of Warwick, and is being led by Associate Professor Andrew Thompson and Professor Jason Madan. The trial is funded by the National Institute for Health Research and is being sponsored by The University of Warwick. The trial has ethical approval from The University of Warwick.

What is the purpose of the trial?

In Australia (University of Monash) researchers have successfully demonstrated that offering online family focused guidance and skills for supporting a child with their mental and emotional wellbeing, has long lasting benefits, fostering a positive and nurturing family environment and helping families develop a wide range of skills and knowledge. The website covers topics such as how to create a positive and nurturing family environment and best support their child’s mental wellbeing.

This leaflet presents independent research funded by the National Institute for Health Research (NIHR) under the Grants for Public Health Research [project number: 17/04/34]. The views expressed in this leaflet are those of the author(s) and not necessarily those of the NIHR or the Department of Health and Social Care.

FUNDED BY NIHR | National Institute for Health Research

The University of Warwick have adapted the website for a UK setting. The PIPA Trial will examine the effectiveness of the online resource in helping support the parents/carers of young people experiencing depression and anxiety.

What will Schools be required to do?

The trial will be a collaboration with secondary schools in the UK, who will work with the research team to engage and recruit parents/carers of young people aged 11-15 years. We will be guided by schools as the best way to engage with their parents/carers. This could be via email, text, school intranet or school events. We hope to collaborate with about 50 schools and recruit 433 families from the UK. We will also invite participating parents and school staff to take part in separate focus groups at a later date to discuss thoughts and experiences of the trial and online resources. This may involve the school aiding the trial team in setting up these focus groups at your school.

Who can take part?

Parents/carers of a young person aged 11-15 years (including non-biological parents, grandparents and legal guardians). They must be able to provide informed consent for both themselves and their young person, have sufficient understanding and knowledge to engage with content presented in English, have access to the internet and a personal email account (for email communication) and mobile phone number (for phone and text messaging communication), and live with the participating young person.

The young person will need to give assent to participate in the trial, have a reading age of 11+ years, have access to a mobile phone for text messaging communication and the internet for questionnaire completion.

In addition participants will need to ensure that they have not been previously entered into the trial, withdrawn from the trial or taken part in an alternative parenting intervention in the last 90 days. If a parent/carer and young person is already participating in the trial, another family dyad from the same household will not be able to register to take part.

Eligibility

If the above criteria are met, and families and young people are happy to take part, they will find out if they are eligible to continue with the trial once their young person has completed eligibility questions about emotional wellbeing.

What will eligible parents/carers/young people be required to do?

The PIPA trial is conducted online so parents/carers or young people will not be required to meet with researchers.

There will be two groups in the trial: one receiving a standard educational package and one receiving a personalised programme. Parents/carers will be randomly selected to one of the groups by computer. Parents/carers will know which group they are in once they have registered on the 'Partners in Parenting' website and completed some questions on their parenting style.

This leaflet presents independent research funded by the National Institute for Health Research (NIHR) under the Grants for Public Health Research [project number: 17/04/34]. The views expressed in this leaflet are those of the author(s) and not necessarily those of the NIHR or the Department of Health and Social Care.

FUNDED BY NIHR | National Institute for Health Research

- ***The Partners in Parenting Website***
  The Partners in Parenting website houses the personalised programme and the standard educational package. Parents/carers will logon to the Partners in Parenting website with their own individual login and will be presented with their individual dashboard.

- ***The Personalised programme***
  A maximum of nine modules will be available in the programme but parents/carers will be able to tailor it by deselecting or selecting additional modules, as required, and dependent on their particular needs. The nine modules include illustrations, audio clips, goal-setting exercises and an end-of-programme quiz. Parents/carers will receive automated feedback at each stage of the programme to consolidate their learning and highlight areas of strength and those with which they may be struggling, with ongoing guidance for improvement.

- ***The Standard Educational Package***
  Parents/carers allocated to this group will receive five factsheets about adolescent development and mental and emotional wellbeing. Factsheets will be accessed by logging in to their personal dashboard. The factsheets provide general information for parents/carers and are designed to represent a selection of resources that are available to parents as part of the current UK health promotion approach for adolescent wellbeing. (The materials were adapted from highly credible existing resources provided on the 'Raising Children Network' website).

Baseline and follow-up questions will include questions about mental wellbeing, depression, anxiety, quality of life and use of health services. Information about age, gender and ethnicity will also be collected at the start of the trial.

More than one parent/carer is welcome to work through the programme. However, we will ask that only one parent/carer completes the online questionnaires. This should be the parent/carer who has given consent.

All parents/carers will receive fortnightly calls from the research team to provide support (such as help with using the Partners in Parenting website) as needed. In alternating weeks, they will receive a text message reminder to continue with their factsheets or modules.

Each set of questionnaires throughout the trial (at baseline, six months and 15 months), should take on average about one hour to complete. This could take slightly longer depending on their answers to some of the questions.

Recruitment for the PIPA trial will finish in early 2023 so parents/carers who would like to take part must register before then.

We will also contact some parents/carers and young people at a later date to invite them to take part in a focus group and/or interview about their experience of the trial.

What are the benefits for schools?

- Help enhance schools' relationship with parents/carers, enabling them to improve their child's mental and emotional wellbeing through an evidence based system of support.

This leaflet presents independent research funded by the National Institute for Health Research (NIHR) under the Grants for Public Health Research [project number: 17/04/34]. The views expressed in this leaflet are those of the author(s) and not necessarily those of the NIHR or the Department of Health and Social Care.

FUNDED BY NIHR | National Institute for Health Research

- Help to develop better resources for young people at risk of developing mental health issues and provide schools and parents/carers with the skills to assist them.
- The opportunity for schools to be an integral part of evidence-based research to improve youth mental health and wellbeing.

What are the possible risks for parents/carers, young people and schools?

Previous literature suggests that young person or parent/carer distress following participation in assessment of mental and emotional wellbeing is unlikely in this population.

Occasionally, however, some participants may find certain topics covered upsetting. We will monitor for any distress and internalised stigma, therefore, throughout the trial. The research team will also monitor risk through regular phone contact with parents/carers.

Address details for participants will be collected for use in the case of an emergency where the research team may be required to contact emergency services due to concerns for a participant.

As a school, you will not as standard be notified as to which families are participating in the trial. This will only be disclosed in the event of the trial team having concerns about the wellbeing of participants.

Will details collected be kept confidential?

All information collected during the trial will be kept confidential at all times and held in compliance with the Data Protection act 2018 and the General Data Protection Regulation. School contact details will be held at Warwick Clinical Trials Unit for the purpose of the trial team need to contacting you. We will use the schools name and location to acknowledge your participation in our final report to the funders.

Trial documentation and data will be archived for at least ten years after completion of the trial. The PIPA database incorporates all data collection, security and anonymisation of data storage and is subject to University of Warwick data policy.

All data will only be accessible to the research team using assigned logins and passwords. All data will be treated in confidence and will not be disclosed or used for any unrelated purpose (except by prior agreement with the participant or to address specified risks to the participant, researcher or others). If the research team identifies a young person deemed at risk or become concerned about their safety, the team will follow their approved Duty of Care procedure.

What will happen to the data collected?

As a publicly-funded organisation, the University of Warwick have to ensure that it is in the public interest when using personal information from participants. This means that if parents/carers and young people from your school agree to participate we will only use their data in the appropriate ways needed to conduct and analyse the research trial.

We will act as the *data controller* for the trial and are committed to protecting the rights of individuals in line with data protection legislation. The Warwick Clinical Trials Unit will archive trial

This leaflet presents independent research funded by the National Institute for Health Research (NIHR) under the Grants for Public Health Research [project number: 17/04/34]. The views expressed in this leaflet are those of the author(s) and not necessarily those of the NIHR or the Department of Health and Social Care.

FUNDED BY NIHR | National Institute for Health Research

documentation and data for at least 10 years after trial completion. The University of Monash will be a *data processer* for the trial and the Partners in Parenting website which will be used to manage both the personalised programme and the standard educational package and based within a Google cloud in the UK.

Parents/carers will be required to enter personal identifiable information about themselves and their child in order to work through their modules or factsheets and provide consent via an online form. This will be stored securely in a Google Cloud. All other participant data will be collected online via the PIPA database which is subject to the University of Warwick data policy. Access to data will be restricted to authorised personnel.

Parents/carers' and young people's rights to access, change or move their information are limited, as we need to manage their information in specific ways in order for the research to be reliable and accurate. The University of Warwick has in place policies and procedures to keep all data safe.

For further information, please refer to the University of Warwick Research Privacy Notice which is available here: https://warwick.ac.uk/services/sim/privacynotices/research/ or by contacting the Information and Data Compliance Team at GDPR@warwick.ac.uk.

Data Transfer

For the purpose of this trial names, gender and contact details of parents/carers and young people will be shared with individuals from the Clinical Research Networks who will be assisting with making the fortnightly phone calls to participants throughout the trial.

The trial team will also be using a text messaging service called 'Twilio' in order to text fortnightly messages and reminders to participants, therefore, mobile phone numbers and names of parents/carers and young people will be shared with 'Twilio'. Twilio's privacy statement can be found here https://www.twilio.com/legal/privacy.

During the course of the trial data will be transferred between the Partners in Parenting website, the University of Warwick and third parties involved in the scoring of the questionnaires. During this transfer only participant identification numbers will be used and data will be anonymous.

Data Processing and Data Sharing agreements will be in place for the above purposes and will be in accordance with University of Warwick's policies.

Anonymised data may also be used for future research, including impact activities following review and approval by an independent Research Ethics Committee. This will be subject to parent/carer and young person consent at the outset of this research project.

What will you do with the results of the trial?

The trial team will share a brief summary of the trial findings with your school. We will present the findings in a trial report and in relevant journals. Individuals will not be identified in any of the publications.

Who has reviewed the trial?

This leaflet presents independent research funded by the National Institute for Health Research (NIHR) under the Grants for Public Health Research [project number: 17/04/34]. The views expressed in this leaflet are those of the author(s) and not necessarily those of the NIHR or the Department of Health and Social Care.

This trial has been reviewed and given favourable opinion by the University of Warwick's Biomedical & Scientific Research Ethics Committee (BSREC): BSREC 20/19-20.

Who is organising and paying for the trial?

The trial is being co-ordinated by the University of Warwick, and led by Professor Andrew Thompson and Professor Jason Madan. The trial is funded by the National Institute for Health Research - (PHR Project: 17/014/34).

Who can I contact for further information?

If you have any questions about the trial, or your schools involvement, either now or in the future, please contact the trial team using the details below:

Dr Charlotte Connor
Assistant Professor
Warwick Clinical Trials Unit, Warwick Medical School
University of Warwick,
Coventry, CV4 7AL
Email: pipa@warwick.ac.uk
Tel: 02476575078

What if there is a problem?

This trial is covered by the University of Warwick's insurance and indemnity cover. If you have any concerns about this trial, please contact the trial team:

The PIPA Trial Team
Warwick Clinical Trials Unit
Warwick Medical School, University of Warwick
Coventry, CV4 7AL
Email: pipa@warwick.ac.uk

Who should I contact if I wish to make a complaint?

Any complaint about the way you have been dealt with during the trial or any possible harm you, your school, staff, pupils or families might have suffered will be addressed. Please address your complaint to the senior University of Warwick official, who is entirely independent of this trial:

Head of Research Governance
Research & Impact Services
University House, University of Warwick
Coventry, CV4 8UW
Email: researchgovernance@warwick.ac.uk
Tel: 024 7657 5733

Thank you for taking time to read this information leaflet and for considering the trial

This leaflet presents independent research funded by the National Institute for Health Research (NIHR) under the Grants for Public Health Research [project number: 17/04/34]. The views expressed in this leaflet are those of the author(s) and not necessarily those of the NIHR or the Department of Health and Social Care.

Parent & Carer Information Sheet

An online parenting intervention to prevent affective disorders in high-risk adolescents: The PIPA Trial

Investigators: Professor Andrew Thompson, Professor Jason Madan & The PIPA Trial Team

Introduction

You are invited to take part in a research trial. Before you decide to take part, you need to understand why the research is being done and what it would involve for you. Please take the time to read the following information carefully. Talk to others about deciding to take part in the trial if you wish.

Please ask the PIPA team about anything that is not clear or if you would like more information (contact details are available at the end of this document). Please take the time to decide whether or not you wish to take part.

Who is organising and funding the trial?

The trial is being coordinated by the University of Warwick, and is being led by Professor Andrew Thompson and Professor Jason Madan. The trial is funded by the National Institute for Health Research. The trial is being sponsored by The University of Warwick.

What is the trial about?

Difficulties with mental wellbeing in childhood and adolescence can lead to more difficulties in young adulthood and beyond. Providing support before these difficulties occur can have an important impact on outcomes and parents/carers are an important source of support for young people. There is a large amount of evidence for interventions to prevent depression and anxiety in young people which is directly targeted towards young people; however, research into interventions targeting parents/carers is limited.

Researchers at the University of Monash in Australia have successfully developed an online, parent-focused programme (Partners in Parenting) which provides families with either a personalised programme in the form of nine modules or a standard educational package to support their child with their mental wellbeing. The personalised programme cover topics such as how to create a positive and nurturing family environment and help families develop a range of skills and knowledge to support their child's mental wellbeing. The standard educational package consists of factsheets which include information on adolescent development and wellbeing.

This leaflet presents independent research funded by the National Institute for Health Research (NIHR) under the Grants for Public Health Research [project number: 17/04/34]. The views expressed in this leaflet are those of the author(s) and not necessarily those of the NIHR or the Department of Health and Social Care.

The University of Warwick has adapted the Australian Partners in Parenting website for use in the UK. This was adapted following guidance from parents/carers and teachers in the local area. The PIPA trial is exploring the effectiveness of the personalised programme and standard educational package, to see which is best at helping parents to reduce depression and anxiety in their children. The trial will involve approximately 433 families in England.

Why have I been invited?

We are inviting parents/carers of children aged 11-15 years in the UK to participate in the trial. You may have been contacted because your school has chosen to collaborate with us on the trial or you may have seen the trial promoted elsewhere.

What would taking part involve?

The diagram below shows what taking part will involve.

Interested parents/carers will be invited to provide contact details and consent for themselves and their child to take part via the trial website.

↓

If consent from parents/carers is received, young people will be asked if they wish to take part.

↓

If they agree to take part, young people will then be asked to complete a questionnaire about emotional wellbeing to confirm if they are eligible to take part in the trial.

↓

Eligible parents/carers and young people will be invited to complete online questionnaires.

↓

Parents/carers will be randomly selected to receive either the personalised programme or standard educational package.

↓

Parents/carers will be asked to complete a questionnaire on their parenting style.

↓

Parents/carers then have up to 3 months to complete the personalised programme or standard educational package.

↓

Parents/carers and young people will be invited to complete online questionnaires again at six months and 15 months after starting the trial.

<u>Definitions</u>

Randomisation – There will be two groups in the trial: one receiving a standard educational package and one receiving the personalised programme. Parents/carers will be randomly allocated to one of the groups by a computer. Parents/carers will know which group they were placed in once they have registered on the

This leaflet presents independent research funded by the National Institute for Health Research (NIHR) under the Grants for Public Health Research [project number: 17/04/34]. The views expressed in this leaflet are those of the author(s) and not necessarily those of the NIHR or the Department of Health and Social Care.

Partners in Parenting website and completed some questions on their parenting style. Neither parents/carers, nor the researchers are able to choose or influence the group into which they are placed.

Partners in Parenting website– this is used to manage the standard educational package and the personalised programme. *(Please note that both the PIPA database and Partners in Parenting website do not work in Internet Explorer. If you are experiencing difficulties, please use another browser such as Chrome or Edge).*

Standard educational package – five factsheets about adolescent development and wellbeing (one released each week).

Personalised Programme – a set of modules selected for each parent/carer covering up to nine different areas of parenting and family life (one module released each week).

<u>The Trial</u>

- The trial is conducted online so you and your child will not be asked to attend meetings with researchers.

- Baseline and follow-up questionnaires include questions about mental wellbeing, depression and anxiety, quality of life and use of health services. Information about age, gender and ethnicity will also be collected at the start of the trial.

- More than one parent/carer per family is welcome to work through the Partners in Parenting website. However, we do ask that only one parent/carer completes the initial questionnaires, and the same parent/carer will also need to complete the follow-up questionnaires. This same parent/carer would provide consent for the trial.

- You can select either text or email as your preferred method of contact from the trial team. (*If you selected email but have not received any emails, please check your 'junk' or 'spam' folder. You can mark our email address as 'safe' to prevent this from happening again).* The trial team may also call you to provide a reminder if you/your child have not completed the registration process.

- All parents/carers will receive fortnightly calls from the research team to check-in and provide support (such as help with using the Partners in Parenting website) as needed. If we haven't been able to reach you, we will leave a voicemail requesting you to call us back. In alternating weeks, parents/carers will receive a text/email message reminder to continue with their factsheets/modules. You will receive an automated email from the Partners in Parenting website when a new module/factsheet is released.

- Parents/carers and young people will be asked to complete online questionnaires again at six months and 15 months after completing the initial questionnaires (total duration of the trial for each family is 15 months).

- Each set of questionnaires (at baseline, six months and 15 months) should take on average about one hour to complete. This could take slightly longer depending on your answers to some of the questions.

- If you decide to join the trial, please can we ask that you do not discuss the online resources (the

This leaflet presents independent research funded by the National Institute for Health Research (NIHR) under the Grants for Public Health Research [project number: 17/04/34]. The views expressed in this leaflet are those of the author(s) and not necessarily those of the NIHR or the Department of Health and Social Care.

personalised programme and standard educational package) with other parents/carers who may be taking part in the trial? This is so that we can accurately measure the effectiveness of the personalised programme and the standard educational package.

- Recruitment for the PIPA trial will finish in early 2023 so parents/carers who would like to take part must register before then.

- Parents/carers may be invited to attend a focus group with other parents/carers about their experience on the trial at a later date. Around 30 parent/carers and young people may also be invited to individual interviews about their experience on the trial. These focus groups and interviews will be audio recorded, and later typed up, and will be held in accordance with the University of Warwick's data policy. If you are asked to take part in a focus group or interview and you do not want to, this is completely fine and will not affect your time on the trial. At the time of asking, we will give you more information on this so that you can make an informed decision and give consent. We will be approaching participants about this in 2021/22.

- University of Warwick's data policy: https://warwick.ac.uk/services/sim/dataprotection

Who can take part?

If you are interested in taking part, you will need to meet each of the following criteria:

- Parents/carers of young people aged 11-15 years (parent/carers include parents, carers, non-biological parents, grandparents and legal guardians).
- Parent/carer age ≥ 18 years.
- Able to provide informed consent for both the parent/carer and young person.
- Sufficient understanding and knowledge to engage with content presented in English.
- Have access to the internet and a personal email account (for email communication) and mobile phone number (for phone and text messaging communication).
- Lives with the participating child.

Your child will also need to fulfil the following criteria:

- Confirmed assent (agreement) to participate in the trial.
- Has a reading age of 11+ years
- Has access to a mobile phone for text messaging communication and the internet for questionnaire completion.
- Score a certain amount on some screening questions about their wellbeing.

In addition to the above, you will need to ensure that the following does not apply to you:

- Previous unsuccessful entry into this trial.
- Withdrawal from the current trial.
- Participation in an alternative parenting intervention in the last 90 days.
- A parent/carer and young person is already taking part in the trial from your household.

If you meet the criteria and you and your child are happy to take part, your eligibility will be confirmed once

This leaflet presents independent research funded by the National Institute for Health Research (NIHR) under the Grants for Public Health Research [project number: 17/04/34]. The views expressed in this leaflet are those of the author(s) and not necessarily those of the NIHR or the Department of Health and Social Care.

your child has completed the eligibility questions about wellbeing. If your child is eligible, you will both be invited to continue with the trial.

What if my child or I am not eligible?

If you are not eligible you will not be able to take part in the trial. If your child is not eligible, you will both be informed that you are ineligible for the trial.
Due to the eligibility requirements for the trial, if after you have entered some information about yourself and your child onto our database and you find out you are ineligible, we will need to keep some identifiable information on you both on our database to ensure that you are not screened for the trial twice. This identifiable data will be deleted once recruitment for the trial ends (early 2023).

Do I have to take part?

No. Taking part in this trial is voluntary and choosing not to take part will not affect you or your child in any way. You can also choose to withdraw at any time, without giving a reason, by contacting one of the research team. Further details about withdrawing from the trial are provided later on in this document.

What are the possible benefits of taking part in this trial?

Taking part in this trial should help improve your knowledge of mental wellbeing in young people and assist you in picking up some tips to help support your child. By participating in this trial you will also be contributing to the evidence for research into adolescent mental health and preventive interventions. The findings of this trial may help to develop better services for young people at risk of developing mental health difficulties and provide parents/carers with skills to assist them.

What are the possible disadvantages, side effects or risks, of taking part in this trial?

Risks in taking part are minimal. It is possible that participants may find some of the topics covered in the trial upsetting. The research team will be able to monitor this through the regular phone contact and provide the necessary support and if needed let you know about where you can go to get further information and advice. If the research team becomes concerned about the welfare of a participant, we will follow approved procedures in dealing with this. This will likely involve a phone call from the team to the parent/carer/school.

Expenses and payments

Each participating family will receive £25 voucher at the end of the trial if questionnaires are completed at each time point (baseline, six months & 15 months) by both parent/carer and young person. This is as an acknowledgement of the time commitment required to complete the questionnaires (around one hour each per time point). This payment will be in the form of a voucher and the trial team will be in touch to arrange this after the completion of the last questionnaire. We will need your address to send this to you.

Will my taking part be kept confidential?

Information collected during this trial will be kept confidential and handled and stored in accordance with the Data Protection Act 2018 and the General Data Protection Regulation. Personal identifying information will be held securely at Warwick Clinical Trials Unit. This includes names and contact details which will be required for communication throughout the trial. Access to this data will be monitored throughout the trial. At the end of the trial (after the last participant has completed their last questionnaire and any interviews

This leaflet presents independent research funded by the National Institute for Health Research (NIHR) under the Grants for Public Health Research [project number: 17/04/34]. The views expressed in this leaflet are those of the author(s) and not necessarily those of the NIHR or the Department of Health and Social Care.

have been completed), names and contact details will be deleted from our records.

You and your child will be assigned participant identification numbers, which will be used during the trial by the PIPA trial team. Examples of when this will be used are when transferring data and for safety monitoring. The reason for having the identification number is to remove the need to transfer and use personal identifiable data outside of the research team. More information on data transfers can be found below.

All data will be treated in confidence and will not be disclosed or used for any unrelated purpose (except by prior agreement from the parent/carer or to address concern/risk to the participant, researcher or others).

If the research team identifies a participant deemed at risk or becomes concerned about their safety, the team will follow University of Warwick-approved procedures and inform the parents/carers, school, clinicians at Warwick Clinical Trials Unit and/or relevant authorities which could include the emergency services.

For the purpose of this trial your name and contact details may be shared with individuals from Clinical Research Networks who will be assisting with making the fortnightly phone calls to participants throughout the trial. The Clinical Research Networks fund and manage infrastructure to support high-quality health and care research in England.

Your child's school will not be made aware of any participation in the trial, except for any circumstances where we may be concerned about a participant's safety.

What will happen to the data collected about me?

As a publicly-funded organisation, the University of Warwick must ensure that it is in the public interest when we use personal identifiable information from people who have agreed to take part in research. This means that if you agree to take part in a research trial, such as this, we will use your data in the ways needed to conduct and analyse the research trial.

We will act as the data controller for the trial. We are committed to protecting the rights of individuals in line with data protection legislation. The Warwick Clinical Trials Unit will archive trial documentation and data for at least 10 years after completion of the trial. This will not include your names and contact details.

The University of Monash will be a data processer for the trial and the Partners in Parenting website (where you will enter some personal identifiable information about yourself and your child, and work through your personalised programme/standard educational package) will be managed by them and based within a Google cloud in the UK.

Parents/carers will be required to provide consent for both themselves and their child via an online form, alongside contact details for themselves and their child which will be stored securely at the Warwick Clinical Trials Unit. All other participant data will be collected online via the PIPA trial database which is subject to the University of Warwick data policy. Access to data will be restricted to authorised personnel.

Address details for participants will be collected for use in the case of an emergency where the research team may be required to contact emergency services due to concerns for a participant.

Your rights to access, change or move your information are limited, as we need to manage your information in specific ways in order for the research to be reliable and accurate. The University of Warwick has in place policies and procedures to keep your data safe.

This leaflet presents independent research funded by the National Institute for Health Research (NIHR) under the Grants for Public Health Research [project number: 17/04/34]. The views expressed in this leaflet are those of the author(s) and not necessarily those of the NIHR or the Department of Health and Social Care.

For further information, please refer to the University of Warwick Research Privacy Notice which is available here: https://warwick.ac.uk/services/sim/privacynotices/research/ or by contacting the Information and Data Compliance Team at GDPR@warwick.ac.uk.

Data Transfer

For the purpose of this trial your name, gender and contact details may be shared with individuals from Clinical Research Networks who will be assisting with making the fortnightly phone calls to participants throughout the trial. Your child's name and gender will also be shared with them.

The trial team will be using a text and email messaging service called Twilio during the course of the trial. This will help the team to send out the fortnightly text/email messages, Partners in Parenting emails and any reminders for questionnaire completion to participants. For the purpose of text messaging, yours and your child's mobile phone numbers and names will be shared with Twilio. For the purpose of sending emails, your email address and yours and your child's names will be shared with Twilio. We will ensure that this data is deleted at the end of the trial (the end of 2024). Twilio's privacy statement can be found here https://www.twilio.com/legal/privacy.

During the course of the trial, data will be transferred from the Partners in Parenting website site to The University of Warwick and to and from the University of Warwick and third parties (including Monash University in Australia) involved in the assessment of the questionnaires. When transferring the Partners in Parenting data and questionnaire data, your participant identification numbers will be used and the data will be anonymous.

Data Processing and Data Sharing agreements will be in place for the above purposes. Data will be transferred securely and in accordance with University of Warwick's policies.

Your anonymised data may also be used for future research, including impact activities following review and approval by an independent Research Ethics Committee and subject to your consent at the outset of this research project.

What will happen if I don't want to carry on being part of the trial?

Taking part in this trial is entirely voluntary and you are free to withdraw from the trial at any point without providing a reason, by contacting the research team using the contact details at the end of this document. Withdrawing from the trial will not affect you or your child in any way.

The eligibility requirements for the trial state that we cannot allow previously ineligible or withdrawn participants enter the trial again and so because of this, we will also need to keep some identifiable information about you and your child on our database to ensure that you are not entered into the trial more than once. These identifiable data will be deleted once recruitment for the trial ends (early 2023). If you choose to withdraw, we will use any data that you have provided us with up to that point.

To safeguard your rights, we will use the minimum personal-identifiable information possible and keep the data secure in line with the University's Information and Data Compliance policies.

If you choose to withdraw, you can either withdraw from the Partners in Parenting website (Personalised Programme or standard educational package) but still complete follow-up questionnaires, or withdraw from

This leaflet presents independent research funded by the National Institute for Health Research (NIHR) under the Grants for Public Health Research [project number: 17/04/34]. The views expressed in this leaflet are those of the author(s) and not necessarily those of the NIHR or the Department of Health and Social Care.

the trial completely at which point your child will also be withdrawn. Likewise, your child can either withdraw from completing questionnaires or withdraw completely from the trial at which point you (the parent/carer) will also be withdrawn. If you choose to withdraw, we will use any data that you have provided us with up to that point.

What will happen to the results of the trial?

We will present the findings of the trial in a report to the funder and in relevant journals. Individual participants will not be identified in any of the publications. If you wish to find out about the results of the trial after the trial has ended, please contact the research team or go to the trial website (www.warwick.ac.uk/pipa).

Who has reviewed the trial?

This trial has been independently reviewed and given favourable opinion by the University of Warwick's Biomedical & Scientific Research Ethics Committee (BSREC): BSREC 20/19-20. This trial meets the required ethics standards.

Who should I contact if I want further information?

If you have any questions about the trial, or your involvement in it, either now or in the future, please contact the PIPA trial team using the details below:

Dr. Charlotte Connor – Assistant Professor
Warwick Clinical Trials Unit, Warwick Medical School
University of Warwick,
Coventry, CV4 7AL
Email: pipa@warwick.ac.uk
Tel: 02476575078

What if there is a problem?

This trial is covered by the University of Warwick's insurance and indemnity cover. If you have any concerns about this trial, please contact the trial team:

The PIPA Trial Team
Warwick Clinical Trials Unit
Warwick Medical School, University of Warwick
Coventry, CV4 7AL
Email: pipa@warwick.ac.uk

Who should I contact if I wish to make a complaint?

Any complaint about the way you have been dealt with during the trial or any possible harm you might have suffered will be addressed. Please address your complaint to the person below, who is a senior University of Warwick official, entirely independent of this trial:

Head of Research Governance

Research & Impact Services, University House

This leaflet presents independent research funded by the National Institute for Health Research (NIHR) under the Grants for Public Health Research [project number: 17/04/34]. The views expressed in this leaflet are those of the author(s) and not necessarily those of the NIHR or the Department of Health and Social Care.

University of Warwick
Coventry, CV4 8UW
Email: researchgovernance@warwick.ac.uk
Tel: 024 7657 5733

If you wish to raise a complaint on how we have handled your personal data, you can contact our Data Protection Officer, Information and Data Director who will investigate the matter: DPO@warwick.ac.uk.

If you are not satisfied with our response or believe we are processing your personal data in a way that is not lawful you can complain to the Information Commissioner's Office (ICO): casework@ico.org.uk

Thank you for taking the time to read this Parent & Carer Information Sheet

This leaflet presents independent research funded by the National Institute for Health Research (NIHR) under the Grants for Public Health Research [project number: 17/04/34]. The views expressed in this leaflet are those of the author(s) and not necessarily those of the NIHR or the Department of Health and Social Care.

Young Person Information Sheet

An online parenting intervention to prevent affective disorders in high-risk adolescents: The PIPA Trial

Investigators: Professor Andrew Thompson, Professor Jason Madan & The PIPA Trial Team

Introduction

You are invited to take part in The PIPA Trial, which is looking at preventing anxiety (feeling worried and scared) and depression (feeling sad) in young people. Before you decide whether you want to take part, you need to understand why this research trial is being done and what you would have to do. Please take the time to read this information carefully and talk to others about deciding to take part in the trial if you would like.

If you have any questions, or would like more information, there are details of who to contact at the end of this information sheet.

What are the possible benefits of taking part in this trial?

Taking part in this trial will help you and your family find out more about mental health and emotions. The results of this trial could help create better ways of helping young people and their families with their mental health.

What is the PIPA research about?

We think the internet is a good way to teach people how to deal with things like mental health. We have been working with researchers in Australia (at Monash University) on the 'Partners in Parenting' website to help parents/carers and young people with their mental health. We are trying to find out whether a personalised programme of nine online modules about parenting or a standard educational package which offers five factsheets, is a good way to help families learn about mental health. Over 400 families in England will take part in the PIPA research study.

Who is organising and funding the trial?

PIPA is being organised by the University of Warwick and led by Professor Andrew Thompson and Professor Jason Madan. It is funded by the National Institute for Health Research, has been reviewed and approved by

This leaflet presents independent research funded by the National Institute for Health Research (NIHR) under the Grants for Public Health Research [project number: 17/04/34]. The views expressed in this leaflet are those of the author(s) and not necessarily those of the NIHR or the Department of Health and Social Care.

the University of Warwick's Biomedical & Scientific Research Ethics Committee (BSREC): BSREC 20/19-20 and is being sponsored by the University of Warwick.

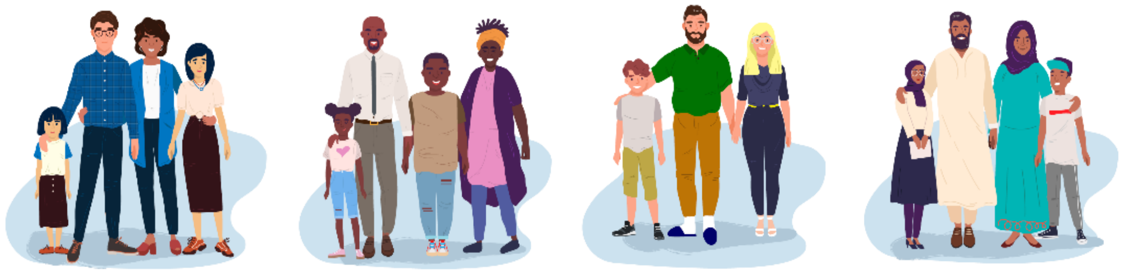

What would taking part involve?

We are inviting families of young people aged 11-15 years in the UK to be involved. The chart below shows what you will have to do if you agree to take part.

We will need to ask your parent/carer if they are happy for your family to take part in the trial.

↓

We will check with you if you are happy to take part.

↓

We will ask you to fill in a short online questionnaire about your emotions to check that you can take part.

↓

We will invite you and your parent/carer to fill in your online baseline questionnaires. This should take you about 30 minutes. A link will be sent to you which will take you to your questionnaires.

↓

Your parent/carer will then start working through either the personalised programme or standard educational package.

↓

We will invite you and your parent/carer to fill in some more online questionnaires again at 6 and 15 months after you filled in the first set of questionnaires. These will also take about 30 minutes to complete. As a thank you, a £25 voucher will be sent to your family for taking part.

This leaflet presents independent research funded by the National Institute for Health Research (NIHR) under the Grants for Public Health Research [project number: 17/04/34]. The views expressed in this leaflet are those of the author(s) and not necessarily those of the NIHR or the Department of Health and Social Care.

FUNDED BY NIHR | National Institute for Health Research

- The online questionnaires will ask about your emotions and mental health and we will also ask about your age and gender. *(Our online questionnaires won't work in Internet Explorer. If you have any problems, you might need to try a different browser like Chrome or Edge).*

- Recruitment for the trial will finish in early 2023 so families that would like to take part must register before then.

- Around 30 parents/carers and young people will also be invited to do an interview about what it was like to take part in the trial. These interviews will be recorded and kept according to the University of Warwick's rules on managing personal information. If you and your family are asked to take part in an interview but you don't want to, this is completely fine and won't affect you taking part in the trial. We will be asking parents/carers and young people about this in 2021/22.

Who can take part?

All of the information we will gather through PIPA will be online so you won't need to go to any meetings with the researchers. There are some things, however, that you must be able to say yes to, if you are to take part in the study:

- Are you between 11 and 15 years old?
- Has your parent/carer said that they are happy for your family to take part?
- Are *you* happy to take part?
- Do you understand what PIPA is about and what you would have to do?
- Do you have a mobile phone so we can text you?
- Do you have access to the internet to fill in the questionnaires?

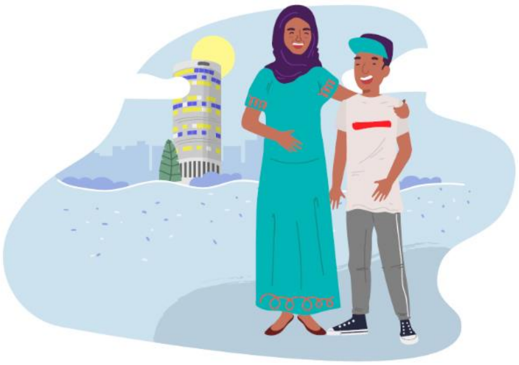

You will also need to score a certain amount on an online questionnaire about your emotions. This is to make sure that the PIPA Trial is suitable for you and your family.

What if I can't take part in the trial?

If you have answered NO to any of the above questions, or score a certain amount on the online questionnaire about your emotions, then, unfortunately, you won't be able to take part in the trial and we will let you and your parent/carer know.

If you have already given us some information about yourself and then find out that you can't take part, we will need to keep some information about you and your parent/carer. This is to make sure that you are not entered into the trial more than once. We will delete all this information once we stop inviting people to take part in the trial (early 2023).

Do I have to take part?

No, you don't have to take part in the trial if you don't want to, this won't affect you or your family in any

This leaflet presents independent research funded by the National Institute for Health Research (NIHR) under the Grants for Public Health Research [project number: 17/04/34]. The views expressed in this leaflet are those of the author(s) and not necessarily those of the NIHR or the Department of Health and Social Care.

FUNDED BY NIHR | National Institute for Health Research

way. If you do decide to take part in the trial but change your mind later on, this is also completely fine. You just need to let the research team know and you don't have to give a reason. More information about this is given later on in this sheet.

What are the possible disadvantages, side effects or risks, of taking part in PIPA?

We have designed PIPA very carefully in order to minimise any disadvantages, however, there is a small chance that you may find some topics in the online questionnaires upsetting. If this happens you should speak to your parent/carer/a member of staff at your school. The research team will also be able to support you and your family by signposting to useful information. If the research team becomes concerned about you, we will follow guidelines on how to deal with this. This will likely involve a phone call from the team to your parent/carer/school.

Expenses and payments

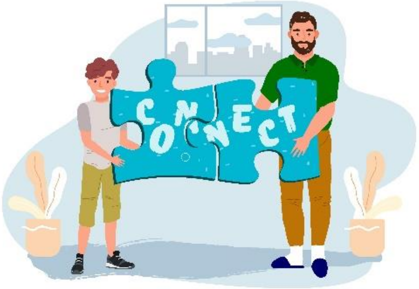

Each family that takes part in the trial will get a £25 voucher at the end of the trial if both parent/carer and young person fill out questionnaires at the start and after 6 and 15 months. This is to say thank you for taking part.

Will my taking part be kept private?

Information we collect about you and your parent/carer will be kept private and we will follow rules to keep this safe. This will only be used to help us run the trial and we won't use this information for anything else without your permission.

Details about your gender, name, address and mobile phone number will be stored at Warwick Clinical Trials Unit. There will be also be some people helping us to make phone calls to parents/carers during the trial and your name and gender will be shared with them. When we've finished texting and phoning all families at the end of the trial (around the end of 2024), we will safely delete your name and contact details.

We need your contact details and address in case the trial team is worried about you and/or your parent/carer and is only collected in the unlikely event that we need to contact you or let other people know. This could include your parent/carer, school, doctors at Warwick Clinical Trials Unit and/or the emergency services.

You and your parent/carer will be given a code, made up of letters and numbers, which we will use as much as we can during the trial, instead of using your names. For example, there will be some times on the trial where other teams of people need to check that the information we are collecting is ok. We will use this code, instead of your name, so that they won't know who you are. These teams are used to checking this sort of information and this happens on other trials too.

Your school will not be told if you and your parent/carer decide to join the trial, except if we are worried about you.

This leaflet presents independent research funded by the National Institute for Health Research (NIHR) under the Grants for Public Health Research [project number: 17/04/34]. The views expressed in this leaflet are those of the author(s) and not necessarily those of the NIHR or the Department of Health and Social Care.

FUNDED BY NIHR | National Institute for Health Research

What will happen to the information collected about me?

The University of Warwick needs to make sure that it is in the public interest when we collect the personal information of people who take part in research. We will only use your information to help us run the trial and not for any other reason.

The University of Warwick will act as the 'data controller'. This means that we are responsible for your information and will follow rules to help us make sure it is kept safe. Warwick Clinical Trials Unit will keep all documents and participant information for at least 10 years after the trial is finished. All trials need to keep such information for a while after trials have finished. This won't include your names and contact details.

Monash University (in Australia) will be a 'data processer' for the trial and the 'Partners in Parenting' website (where your parent/carer will enter information about themselves and you) will be managed by them and kept safe in a Google cloud in the UK.

All other information we collect about you and your parent/carer will be collected via the PIPA Trial database. Only people working on the PIPA Trial will be able to log into this database.

If you give us some information and then ask us to change it, we might not be able to. This is because we need to follow rules on managing information and manage it in specific ways to keep it reliable and correct.

For further information, please follow the link to the University of Warwick Research Privacy Notice which is available here: https://warwick.ac.uk/services/sim/privacynotices/research/ or by contacting the Information and Data Compliance Team at GDPR@warwick.ac.uk.

University of Warwick's rules about managing information: https://warwick.ac.uk/services/sim/dataprotection

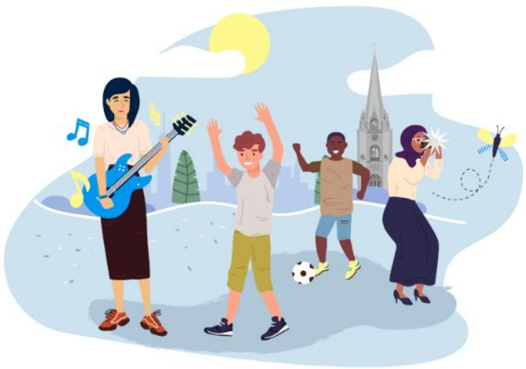

Sharing information

We will be using a text messaging service called 'Twilio' during the trial to help send out reminders to you to complete questionnaires. Your name and mobile phone number will be shared with 'Twilio' but this information will be deleted at the end of the trial (2024). Twilio's privacy statement can be found here https://www.twilio.com/legal/privacy.

If information is shared between the University of Warwick and other companies about the online questionnaires you fill in we will use your code and NOT your name, and the information will be kept anonymous. The only time your name and gender will be shared is with the people helping us to make the phone calls/texts during the trial and if we are ever worried about you.

We have data sharing agreements for all these reasons and information will be shared according to University of Warwick rules. We may use your anonymised information (not your name) for future research projects if your parent/carer lets us know at the start of the trial that this is okay.

This leaflet presents independent research funded by the National Institute for Health Research (NIHR) under the Grants for Public Health Research [project number: 17/04/34]. The views expressed in this leaflet are those of the author(s) and not necessarily those of the NIHR or the Department of Health and Social Care.

FUNDED BY NIHR | National Institute for Health Research

What will happen if I don't want to carry on being part of the trial?

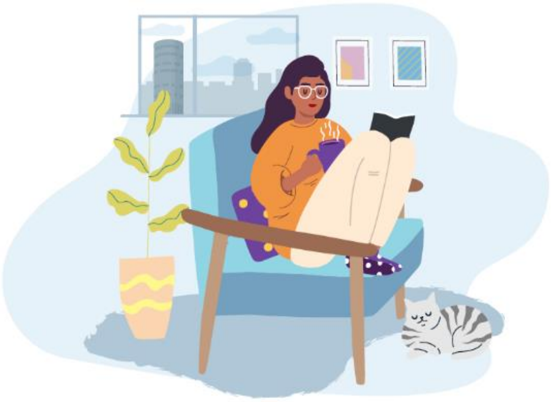

Taking part in this trial is entirely up to you and you can stop taking part at any point without giving a reason and this won't affect you in any way. If you choose to withdraw, we will use any data that you have given us up to that point. Your parents/carers, however, can carry on working through the online resources if they want to. If you would like your parent/carer to stop completing the questionnaires about you, you can contact us and we will let them know that they will have to stop taking part.

What will happen to the results of the trial?

We will write up the findings of this trial in a report and in some educational journals. You and your parent/carer will not be named in any of the publications. If you would like to find out about the results of PIPA after it has ended, please contact the research team on the email given at the end of this information sheet.

Who should I contact if I want more information?

If, at any point during the trial, you have any questions, please contact the PIPA trial team using the details below:

Dr. Charlotte Connor – Assistant Professor
Warwick Clinical Trials Unit, Warwick Medical School
University of Warwick,
Coventry, CV4 7AL
Email: pipa@warwick.ac.uk
Tel: 02476575078

What if there is a problem?

This trial is covered by the University of Warwick's insurance and indemnity cover. If you have any worries about this trial, please contact the trial team:

The PIPA Trial Team
Warwick Clinical Trials Unit
Warwick Medical School, University of Warwick
Coventry, CV4 7AL
Email: pipa@warwick.ac.uk

Who should I contact if I want to make a complaint?

Any complaint about the way you have been dealt with during this trial or any possible harm you might have suffered will be addressed. Please address your complaint to the person below, who is a senior University of Warwick official, entirely independent of this trial.

This leaflet presents independent research funded by the National Institute for Health Research (NIHR) under the Grants for Public Health Research [project number: 17/04/34]. The views expressed in this leaflet are those of the author(s) and not necessarily those of the NIHR or the Department of Health and Social Care.

Head of Research Governance
Research & Impact Services, University House
University of Warwick
Coventry, CV4 8UW
Email: researchgovernance@warwick.ac.uk
Tel: 024 7657 5733

If you would like to raise a complaint on how we have handled your personal information, you can contact our Data Protection Officer, Information and Data Director who will investigate the matter: DPO@warwick.ac.uk.

If you are not satisfied with our response or believe we are processing your personal information in a way that is not lawful you can complain to the Information Commissioner's Office (ICO): casework@ico.org.uk

Thank you for taking the time to read this Young Person Information Sheet

This leaflet presents independent research funded by the National Institute for Health Research (NIHR) under the Grants for Public Health Research [project number: 17/04/34]. The views expressed in this leaflet are those of the author(s) and not necessarily those of the NIHR or the Department of Health and Social Care.

PIPA

An online **P**arenting **I**ntervention to **P**revent affective disorders in high-risk **A**dolescents:
The PIPA Trial

The PIPA Trial

The PIPA trial is exploring the effectiveness of an online resource for parents/carers to help reduce depression and anxiety in young people.

Who can take part?

With the assistance of schools, we will be recruiting families of young people aged 11-15 from the UK.

What does it involve?

Parents/carers and young people will be asked to fill in some online questionnaires and parents/carers will be invited to work through a series of online modules or factsheets.

Benefits for families

Improve knowledge of mental health and emotional wellbeing in young people. Pick up some useful tips and strategies to help support young people.

Get in touch!

Email: PIPA@warwick.ac.uk
Phone: 02476574316 or 02476575078
Website: www.warwick.ac.uk/pipa

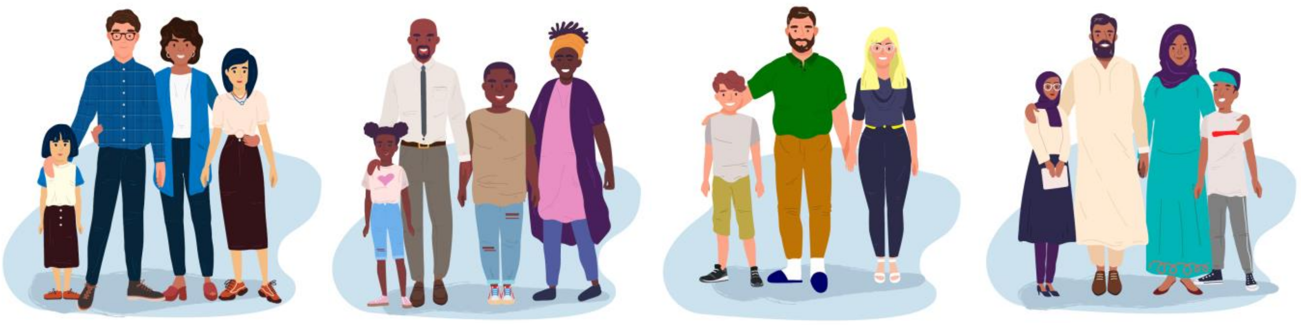

WARWICK
CLINICAL TRIALS UNIT

V2.0, Info poster, 31.08.2021
ISRCTN63358736
