# Supplementary material for: An online parenting intervention to prevent affective disorders in high-risk adolescents: the PIPA trial protocol
Source: Trials. 2022 Aug 15;23:655. doi: 10.1186/s13063-022-06563-8 (PMC9376903; doi:10.1186/s13063-022-06563-8)
Supplement: Supplementary file 2 — Additional file 2. Process evaluation focus group question frameworks. [file 13063_2022_6563_MOESM2_ESM.pdf]

Process Evaluation Focus Group – Question Frameworks

1. Teachers

Focus Group - Teachers		
**The Trial**		**Questions**
**Context**	**Factors which shape how trial might work or which might affect/impact the trial**	1. Why did you take part in the PIPA Trial?
		2. How easy/difficult was it to promote the PIPA trial in your school?
		3. How easy/difficult was it to encourage families to participate in the PIPA trial?
**Implementation**	**The structure through which the trial was delivered, quality, quantity**	4. What worked well/didn't work well in engaging families to take part in the PIPA trial?
		5. What worked well/didn't work well in terms of working with the PIPA trial team?
		6. Did COVID-19 restrictions impact on engaging and recruiting families into the trial? If so how?
		7. How did you promote the PIPA trial in your school?
		8. In what ways did you encourage families to participate in the PIPA trial?
**Impact**	**Trial activities, participant interaction and changes**	9. Can you think of any benefits or disadvantages for your families from being involved in a research trial? In what ways?
		10. Have you had any feedback from families who took part in the PIPA Trial?
		11. Have you had any feedback from families who were interested in taking part in the PIPA trial but were not eligible?
		12. Would you and your school consider taking part in a research trial in the future?

**The Parenting Programme**		
**Context**	**Factors which shape how personalised programme and factsheets might work or be affected**	13. The trial was designed so that parents could complete their parenting programmes online and in their own time. Did this influence the school's decision to take part in the trial?
**Implementation**	**The structure through which the personalised programme and factsheets were delivered, quality, quantity**	14. Do you think that online personalised and factsheet programmes are a good idea? If so why? If not, why not
		15. Can you think of any benefits or disadvantages for your families from taking part in the parenting programmes?
		16. If so, in what ways?
**Impact**	**Intervention activities, participant interaction and changes**	17. Has there been a change in your relationship with families after enabling them to take part in a personalised or factsheet programme?
		18. Would you recommend engaging in a research trial to other schools?

2. Parents/Carers

Focus Group – Parents/Carers		
**The Trial**		**Question**
**Context**	**Factors which shape how trial might work or which might affect/impact the trial**	1. How did you find out about the PIPA trial and why did you take part?
		2. Were you given enough information about the Trial before deciding to take part? What other information would you have liked?
		3. How easy/difficult was it to encourage your child to participate with you in the PIPA trial?
		4. What worked well/didn't work well in terms of working with the PIPA trial team
**Implementation**	**The structure through which the trial was delivered, quality, quantity**	5. Can you think of any benefits or disadvantages for you or your child from being involved in a research trial? In what ways?
		6. Would you consider being involved in a trial again? If so, why? If not, why?
**Impact**	**Trial activities, participant interaction and changes**	7. Did COVID-19 restrictions impact on your decision to take part in the trial or not?
**The Parenting Programme**		**Question**
**Context**	**Factors which shape how personalised programme and factsheets might work or which might affect/impact the intervention**	8. Do you think that the programme you received was suitable and appropriate for all ethnicities and socio-economic groups?
		9. Do you think that the length of the programme you received was too long/too short?
		10. Did you manage to access and complete all of your modules or factsheets easily? If not, why?
		11. How did you feel about the online/in your own time aspect of the programme?

**Implementation**	**The structure through which the personalised programme and factsheets were delivered, quality, quantity**	12. Did you find the programme you received engaging? Videos/audio/illustrations/information? If not, why not?
		13. Did you enjoy working through your programme? If not, why not?
		14. Do you think you and your family have benefited from taking part in your parenting programme? If so, in what ways?
		15. Do you think that you have learned new skills and strategies from your parenting programme that you will use in the future? If so, what were they? If not, why?
		16. Do you think that the programme was especially relevant in the current COVID pandemic? Did the COVID restrictions affect your ability to implement any new skills or strategies from the programme?
**Impact**	**Intervention activities, participant interaction and changes**	17. Do you think that your child found the programme you received helpful or unhelpful?
		18. Was it easy to engage your child in the programme you received?
		19. Do you think that online personalised parenting programmes are a good idea? If so, why? If not, why not?
		20. What was your overall impression of the personalised programme/factsheet programme you received?
		21. Would you recommend the programme you received to other parents/carers?
