# Supplementary material for: An online parenting intervention to prevent affective disorders in high-risk adolescents: the PIPA trial protocol
Source: Trials. 2022 Aug 15;23:655. doi: 10.1186/s13063-022-06563-8 (PMC9376903; doi:10.1186/s13063-022-06563-8)
Supplement: Supplementary file 3 — Additional file 3. Process evaluation family interview question framework. [file 13063_2022_6563_MOESM3_ESM.pdf]

Process Evaluation Family Interviews – Question Framework

Family Dyad Interviews							
Trial							
Context							
Q1 (parent/carer & child)							
Sociodemographic details	Family role (Mother, Father.Daughter etc.,)	Ethnic heritage Asian/Asian British, Black/African/Caribbean/Black British, Mixed ethnic background, White, Other ethinc group (please specify), Prefer not to answer	Age range 18-24, 25-34, 35-44, 45-54, 55-64, 65 & over, Prefer not to answer	Occupation<br><br>Prefer not to answer			
Q2 (parent/carer & child)							
Was there anything that influenced your decision to take part in the trial?	Did you discuss it with anyone else?						
Q3 (parent/carer & child)							
How did you both first find out about the trial?	School/teacher etc.,	Newsletter/email etc.,					

Process Evaluation Family Interviews – Question Framework

Q4 (parent/carer & child)							
Had either of you taken part or been invited to take part in a research trial through your school before?	Yes	What research was that?					
	No						
**Implementation**							
Q5							
When your school got in touch with you about the trial did they provide you both with enough information to make an informed decision about the trial?	Yes	What did they give you?	Did they explain to you why they thought it was a good idea to take part in the trial?	Did their explanations influence your decisions to take part?			
	No	Why not?	What would you have liked to have had explained?				
**Impact**							
Q6 (parent/carer & child)							
Were both you and your child happy to take part in the trial?	Yes						

	No	Why was this?	Who was the most keen?				
Q7							
Would you both like to take part in a research trial again?	Yes	Why?	What did you particularly enjoy about taking part in the trial?				
	No	Why?	Was there anything in particular that you did not enjoy about being part of a trial?				
**Parenting Programme**							
**Implementation**							
Q8 (parent/carer – personalised programme group only)							
At the start of the trial you completed a parenting questionnaire, did you receive feedback on your parenting style?	Yes	How useful did you find the feedback?	What did you learn or gain from this feedback?				
Q9 (parent/carer)							

Process Evaluation Family Interviews – Question Framework

What programme did you receive?	Personalised programme	How many modules were recommended to you?	Did you access all of these modules/edit them/choose your own?	How useful were the modules?	Were there any that particularly useful/unhelpful?	Why?	
	Factsheets	How many factsheets did you access?		How useful were the factsheets?	Were there any that you particularly useful/unhelpful?	Why?	
Q10 (parent/carer & child)							
How did you both feel about being randomly selected to get one programme or the other?							
Q11 (parent/carer)							
How did you access your particular programme?	Personalised Programme	Mobile phone, tablet, computer or laptop or combination	Did you have a preferred way of accessing your modules?	Why was this?	Were they easy to access?	Did you experience any problems accessing your modules?	
	Factsheets	Mobile phone, tablet, computer or laptop or combination	Did you have a preferred way of accessing your factsheets?	Why was this?	Were they easy to access?	Did you experience any problems accessing your factsheets?	
Q12 (parent/carer)							

Process Evaluation Family Interviews – Question Framework

When did you usually access your programme?	Personalised	Why was this?	Did you revisit your modules more than once?	Why was this?			
	Factsheet	Why was this?	Did you revisit your factsheets more than once?	Why was this?			
Q13 (parent/carer)							
Did you find the programme engaging or not engaging?	Personalised	Information, videos, audios, illustrations, quizzes etc., language used	Why was this?	Was there anything that could have been done to improve these?			
	Factsheet	Information, language used, format	Why was this?	Was there anything that could have been done to improve the factsheets?			
Q14 (parents/carer)							
Was the phone call/email/text support from the research team helpful or unhelpful?	Were there other ways of support you would have preferred?						
Impact							
Q15 (parent/carer)							

Process Evaluation Family Interviews – Question Framework

Have you been trying to make changes in your family since participating in your programme?	Personalised	If so, how often are you trying to make these changes? weekly, daily?<br><br>If not, why not?	Do you think this had anything to do with the programme and why?<br><br>Who will benefit from them?	What kind of changes are you trying to make? (related to 9-DOMAINS?)<br><br>Communication<br>Involvement<br>Relationships<br>Rules<br>Conflict<br>Healthy living<br>Stress<br>Anxiety<br>Other	Have these changes been successful? If so, in what ways?<br><br>If not, why?	How confident are you that you will be able to maintain the changes you have made?<br><br>If not, why not?	Did the COVID restrictions get in the way of any changes you wanted to make? If so, which ones and in what ways?
	Factsheet	If so, how often are you trying to make these changes? weekly, daily?<br><br>If not, why not?	What are your reasons for trying to make changes?<br><br>Who will benefit from them?	What kind of changes are you trying to make? (related to factsheet topics)<br><br>Teenage development<br>The developing brain<br>Changing body<br>Resilience<br>Wellbeing	Have these changes been successful? If so, in what ways?<br><br>If not, why?	How confident are you that youwill be able to maintain the changes you have made?<br><br>If not, why not?	
Q16 (child)							
Did you realise your parent/carer was trying to make changes in your family relationship?	How did you feel about this? Did you like the changes they were making?	Did you engage with them to help make these changes?	If yes, in what way?	If not, why not?	What would have made you more likely to engage/help with your		

		Did you think that anything else was going on?			parent/carer in making these changes?		
Q17 (parent/carer)							
Did you feel that your child was aware that you were trying to make changes?	Did they work with you to help make the changes?	If yes, in what ways	If not, why not?	What would have made your child more likely to engage with you in making these changes?			
Q18 (parent/carer)							
Do you think your parenting skills been affected since your took part in your programme?	Personalised	If so, in what ways?<br><br>If not, why not?	Are there skills that you would still like to improve/gain?<br>What are these?				
	Factsheet	If so, in what ways?<br><br>If not, why not?	Are there skills that you would still like to improve/gain?<br>What are these?				
Q19 (child)							
Do you think that your parent/carers behaviour has changed since they took part in their programme?	Personalised	If so, in what ways?<br><br>If not, why not?	Are there skills and behaviours that you would still like your parent to work on?<br>What are these?				
	Factsheets	If so, in what ways?<br><br>If not, why not?	Are there skills that you would still like your parent to work on?<br>What are these?				
Q20 (parent/carer)							

Process Evaluation Family Interviews – Question Framework

Can you describe how helpful or unhelpful your programme was for you as a parent/carer?	Personalised	If so, in what ways?	If not, why not?	What might have made the personalised programme more helpful for you?			
	Factsheet	If so, in what ways?	If not, why not?	What might have made the factsheets more helpful for you?			
Q21 (parent/carer)							
Can you describe how helpful or unhelpful your programme was for your child?	Personalised	If so, in what ways?<br><br>If not, why not?	Do you think that it has helped your child become more resilient?	Do you think it has equipped your child with some skills and strategies to help with them mental and emotional wellbeing?	What might have made the online personalised programme more helpful for your child?		
	Factsheet	If so, in what ways?<br><br>If not, why not?	Do you think it has helped your child become more resilient?	Do you think it has equipped your child with some skills and strategies to help them with their mental and emotional wellbeing?	What might have made the factsheets more helpful for your child?		
Q22 (child)							
Can you describe how helpful or unhelpful the programme was for your parent/carer?	Personalised	If so, in what ways?<br><br>If not, why not?					
	Factsheet	If so, in what ways?					

Process Evaluation Family Interviews – Question Framework

		If not, why not?					
Q23 (child)							
Can you describe how helpful or unhelpful l the programme was for you?	Personalised	If so, in what ways?<br><br>If not, why not?					
	Factsheet	If so, in what ways?<br><br>If not, why not?					
Q24 (parent/carer & child)							
Do you think the programme was especially useful or unhelpful at this time of COVID pandemic?	Personalised	If so, why?<br><br>If not, why not?					
	Factsheet	If so, why?<br><br>If not, why not?					
Q24 (parent/carer & child)							
Was there anything either of you did not like about your programme?	Personalised						
	Factsheet						
Q25 (parent/carer & child)							
Would you recommend your programme to others?	Personalised	If yes, why?<br><br>If not, why?					
	Factsheet	If yes, why?<br><br>If not, why?					

Process Evaluation Family Interviews – Question Framework

