# Supplementary material for: An online parenting intervention to prevent affective disorders in high-risk adolescents: the PIPA trial protocol
Source: Trials. 2022 Aug 15;23:655. doi: 10.1186/s13063-022-06563-8 (PMC9376903; doi:10.1186/s13063-022-06563-8)
Supplement: Supplementary file 4 — Additional file 4. Parent consent form and young person assent form. [file 13063_2022_6563_MOESM4_ESM.pdf]

Parent consent form

	Please note that you must agree to each point below in order to take part in the study.		
1	I understand that only one of my children can take part in this study.	Yes/No	Required field
2	I confirm that my child is aged between 11 and 15 years.	Yes/No	Required field
3	I confirm that my child lives with me.	Yes/No	Required field
4	I confirm that I have access to the internet and a laptop/phone/tablet for this study.	Yes/No	Required field
5	I confirm that I have access to a personal email account and mobile phone number and am happy to receive phone calls and reminder texts from the trial team. I understand a voicemail will be left on my phone if the trial team have been unable to contact me after 3 attempts.	Yes/No	Required field
6	I confirm that I have not participated in a parenting intervention in the last 90 days.	Yes/No	Required field
7	I confirm that I have not previously been screened or randomised for this study.	Yes/No	Required field
8	I confirm that I have read and understood the information sheet (version & date) for the above trial. A link to this can be found on the study website here; (insert link). I have had the opportunity to consider the information, ask questions and have had these answered satisfactorily.	Yes/No	Required field
9	I understand that my participation and my child's participation is voluntary and that we are free to withdraw at any time without giving any reason and without our legal rights being affected.	Yes/No	Required field
10	I understand that data collected during the study will be looked at by individuals from the Warwick Clinical Trials Unit where it is relevant to our participation in this research. I give permission for these individuals to have access to our records.	Yes/No	Required field
11	I understand the reasons for collecting my address and contact details and I am happy to provide these details.	Yes/No	Required field
12	I understand that for the purpose of the study, my name and contact details will be shared with individuals from the Clinical Research Network and I am happy for these details to be shared.	Yes/No	Required field
13	I understand that my child and I will be contacted after 6 and 15 months in order to complete follow-up questionnaires.	Yes/No	Required field
14	I understand that I may be contacted at a future date and invited to an interview and/or focus group about the study experience, and understand that my child will also have the option to attend the interview.	Yes/No	Required field

15	I understand that the information collected about my child and I will be used to support other research in the future, and may be shared anonymously with other researchers.	Yes/No	Required field
16	I agree to participate in this study.	Yes/No	Required field
17	I agree for my child to participate in this study.	Yes/No	Required field
18	If you do not wish to take part, please can you let us know why?	Text box	Not required

Young person assent form

	To be able to take part in the PIPA study, you will need to answer yes to questions 1 to 6		
1	I have read and understand the information sheet (version & date) for this study. A link to this can be found on the study website here; (insert link).	Yes/No	Required field
2	I have had time to think about the information and ask any questions.	Yes/No	Required field
3	I understand that I don't have to take part in this study if I don't want to.	Yes/No	Required field
4	I understand that I can stop taking part in this study at any time.	Yes/No	Required field
5	I understand that after 6 and 15 months I will be asked to answer some more questions.	Yes/No	Required field
6	I am happy to take part in this study.	Yes/No	Required field
7	If you do not want to take part, please can you tell us why?	Text box	Not required
